# PTPN2 links colonic and joint inflammation in experimental autoimmune arthritis

**DOI:** 10.1172/jci.insight.141868

**Published:** 2020-10-15

**Authors:** Wan-Chen Hsieh, Mattias N.D. Svensson, Martina Zoccheddu, Michael L. Tremblay, Shimon Sakaguchi, Stephanie M. Stanford, Nunzio Bottini

**Affiliations:** 1 Department of Medicine, UCSD School of Medicine, La Jolla, California, USA.; 2 Department of Rheumatology and Inflammation Research, Sahlgrenska Academy, University of Gothenburg, Gothenburg, Sweden.; 3 Rosalind and Morris Goodman Cancer Research Centre,; 4 Department of Biochemistry, and; 5 Division of Experimental Medicine, Department of Medicine, Faculty of Medicine and Health Sciences, McGill University, Montréal, Québec, Canada.; 6 Laboratory of Experimental Immunology, Immunology Frontier Research Center, Osaka University, Suita, Japan.; 7 Department of Experimental Pathology, Institute for Frontier Medical Sciences, Kyoto University, Kyoto, Japan.

**Keywords:** Autoimmunity, Inflammation, Arthritis, Autoimmune diseases, Phosphoprotein phosphatases

## Abstract

Loss-of-function variants of protein tyrosine phosphatase non-receptor type 2 (*PTPN2*) enhance risk of inflammatory bowel disease and rheumatoid arthritis; however, whether the association between *PTPN2* and autoimmune arthritis depends on gut inflammation is unknown. Here we demonstrate that induction of subclinical intestinal inflammation exacerbates development of autoimmune arthritis in SKG mice. *Ptpn2*-haploinsufficient SKG mice — modeling human carriers of disease-associated variants of *PTPN2* — displayed enhanced colitis-induced arthritis and joint accumulation of Tregs expressing RAR-related orphan receptor γT (RORγt) — a gut-enriched Treg subset that can undergo conversion into FoxP3^–^IL-17^+^ arthritogenic exTregs. SKG colonic Tregs underwent higher conversion into arthritogenic exTregs when compared with peripheral Tregs, which was exacerbated by haploinsufficiency of *Ptpn2*. *Ptpn2* haploinsufficiency led to selective joint accumulation of RORγt-expressing Tregs expressing the colonic marker G protein–coupled receptor 15 (GPR15) in arthritic mice and selectively enhanced conversion of GPR15^+^ Tregs into exTregs in vitro and in vivo. Inducible Treg-specific haploinsufficiency of *Ptpn2* enhanced colitis-induced SKG arthritis and led to specific joint accumulation of GPR15^+^ exTregs. Our data validate the SKG model for studies at the interface between intestinal and joint inflammation and suggest that arthritogenic variants of PTPN2 amplify the link between gut inflammation and arthritis through conversion of colonic Tregs into exTregs.

## Abstract 2

Loss of protein tyrosine phosphatase non-receptor type 2 amplifies the link between gut and joint inflammation through conversion of colonic Tregs into arthritogenic exTregs.

## Introduction

Genome-wide association studies (GWAS) have identified more than 100 risk genes for rheumatoid arthritis (RA), spondyloarthropathies (SpAs), as well as other forms of immune-mediated arthritis (1–3). The dissection of “causal variants” and of their functional effects at the molecular, cellular, and whole-organism levels is rapidly progressing (4). However, it is believed that functional alterations induced by genetic variants are insufficient per se to trigger common polygenic autoimmune diseases, whose pathogenesis rather depends on a complex interaction between genetically encoded functional variations and nongenetic risk factors, such as environment exposures and/or concurrent local or systemic pathology (5). An important nongenetic risk factor for autoimmune arthritis is gut inflammation, which can be triggered by dysbiosis and/or other mechanisms (“gut-joint axis,” refs. 6–12).

The gut mucosa is enriched with both immune-suppressive “tissue-resident” colonic forkhead box P3^+^ (FoxP3^+^) regulatory CD4^+^ T cells (Tregs) and RAR-related orphan receptor γT^+^ (RORγt^+^) IL-17–producing effector CD4^+^ T cells (Th17) (13, 14). Circulation of T cells between the gut and inflamed joints during arthritis has been reported, and there is supporting evidence for circulation of gut-induced IL-17–producing T cells as a potential pathogenic mechanism in RA (15–18). However, the involvement of intestinal CD4^+^ T cells in the pathogenesis of RA remains largely unknown, and whether intestinal Tregs play a role in inflammatory arthritis has not yet been addressed.

Extensive evidence in multiple mouse models supports the dependence of autoimmune arthritis on the gut microenvironment (9, 18–20). SKG mice, which under specific pathogen–free conditions develop spontaneous Th17-dependent autoimmune arthritis, display no spontaneous disease when maintained in germ-free conditions (21–23). In addition, curdlan-injected SKG mice develop IL-23–dependent small intestine inflammation, gut dysbiosis, and spondyloarthropathy resembling enteropathic arthritis (24). Thus, SKG mice seem to be an ideal model for mechanistic assessment of the gut-joint inflammation axis.

Several arthritis-predisposing genes are shared autoimmunity genes and predispose to inflammatory bowel disease (IBD). The shared autoimmune nature of these genes is at least in part due to their promotion of basic mechanisms of autoimmunity or inflammation (25–27). However, it is reasonable to hypothesize that these shared genetic risk factors might also in part operate by amplifying the reciprocal promotion of clinical or subclinical inflammation between guts and joints. One of the genes that highly associates with RA and IBD encodes for the protein tyrosine phosphatase (PTP) PTPN2, also known as T cell PTP. RA-associated *PTPN2* haplotypic blocks of noncoding single nucleotide polymorphisms (SNPs) also associates with IBD (28–31). Homozygous carriers of the *PTPN2* tagging SNP rs1893217 display a 30%–50% decrease in *PTPN2* mRNA expression in memory CD4^+^ T cells (32). Furthermore, the same rs1893217 risk allele drives reduced PTPN2 protein expression and acts as a loss-of-function variant when transfected into THP-1 cells, a phenotype that is also observed in colonic fibroblasts isolated from carriers of the *PTPN2* tagging SNP rs2542151 (33, 34).

PTPN2 is a ubiquitous PTP that is highly expressed in immune cells (35, 36). The importance of PTPN2 in inflammation is exemplified by the fact that global deletion of this PTP induces myeloid-driven systemic inflammation and lethality in mice (37). Furthermore, T cell–specific deletion of PTPN2 results in development of aggressive colitis and signs of systemic autoimmunity (38, 39). These phenotypes have been attributed to the important role of PTPN2 in regulating activation of Janus kinases (40); signal transducers and regulators of transcription-1 (STAT1), STAT3, and STAT5 (41); as well as T cell receptor (TCR) signaling (38). Consistent with the key role of PTPN2 loss of function in autoimmunity, recent studies have also validated PTPN2 as an important target for cancer immunotherapy (42, 43).

As mentioned, human disease-associated variants of *PTPN2* only induce a partial loss of PTPN2 (32), which can be modeled by studying *Ptpn2*-haploinsufficient mice (44, 45). When compared with the dramatic phenotypes observed in *Ptpn2*-deficient animals, *Ptpn2*-haploinsufficient mice remain healthy under normal conditions (37, 38), but in the presence of inflammatory triggers or when combined with other autoimmune disease–predisposing genes, they can develop gut or joint inflammation (44, 45). This observation is consistent with the idea that disease-associated variants of *PTPN2* need to interact with other risk factors to induce disease in humans.

In human patients with IBD, carriers of the *PTPN2* tagging SNP rs1893217 show enhanced expansion of disease-promoting Th17 and impaired expansion of disease-protective Tregs in colonic tissue (46). Th17 cells are also considered key players in the pathogenesis of RA (47–49). Tregs’ conversion into FoxP3^–^IL-17^+^ exTregs is thought to contribute to the generation of highly autoreactive IL-17^+^ effector T cells in RA (44, 50, 51). We recently showed that *Ptpn2* haploinsufficiency, which models the loss of PTPN2 expression observed in carriers of *PTPN2* risk alleles for IBD and RA, enhances development of Th17-dependent arthritis when introduced onto the SKG mouse background. We identified a novel Treg-intrinsic mechanism for PTPN2 in maintaining peripheral Treg stability during arthritic inflammation and limiting the generation of highly arthritogenic exTregs (44).

In the present study we sought to understand how the interaction between loss of function of *PTPN2* and intestinal inflammation affects the pathogenesis of arthritis in human carriers of IBD- and RA-associated *PTPN2* variants. To model such interaction, we assessed the effect of PTPN2 haploinsufficiency in a potentially new model of arthritis induced by subclinical colonic inflammation in SKG mice.

## Results

### Arthritogenic CD4^+^ T cells are present in the colons of SKG mice.

**Figure 1:**
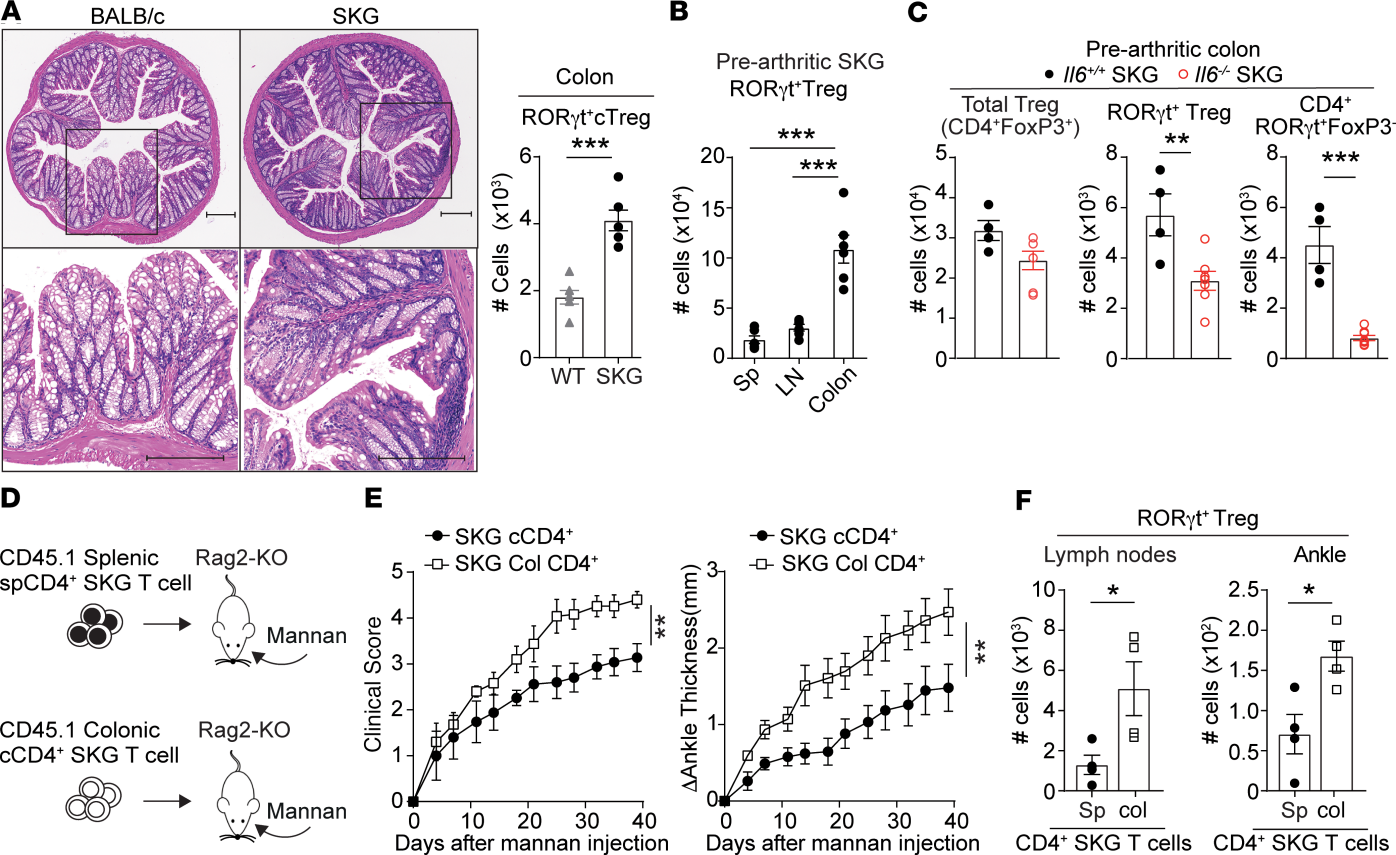
Arthritogenic CD4^+^ T cells are enriched in the colons of SKG mice. (**A**) *Left:* representative H&E staining of colons obtained from 8- to 10-week-old BALB/c (*n* = 4) and prearthritic SKG BALB/c (*n* = 4) mice. Scale bars: 200 μm. *Right:* RORγt^+^ cTregs (CD45^+^TCRβ^+^CD4^+^RORγt^+^FoxP3^+^) in colons isolated from BALB/c and prearthritic BALB/c SKG mice (*n* = 6/group). (**B**) RORγt^+^ Tregs (CD4^+^FoxP3^+^RORγt^+^) in spleen, lymph nodes, and colons of prearthritic SKG BALB/c mice (*n* = 6). (**C**) Number of total Tregs (CD4^+^FoxP3^+^), RORγt^+^ Tregs (CD4^+^RORγt^+^FoxP3^+^) and RORγt^+^FoxP3^–^CD4^+^ T cells (CD4^+^FoxP3^–^RORγt^+^) in the colons of prearthritic female *Il6^+/+^* (*n* = 4) and *Il6^–/–^* (*n* = 7) SKG mice. (**D**) Adoptive transfer of either splenic or colonic CD45.1 CD4^+^ SKG T cells (spCD4^+^ or cCD4^+^, respectively; *n* = 5 mice/group) into Rag2-KO mice. Arthritis was induced 1 week after cell transfer by an intraperitoneal injection of mannan. (**E**) Clinical score and change in ankle thickness. (**F**) Flow cytometric analysis of RORγt^+^ Tregs (CD4^+^FoxP3^+^RORγt^+^) cells in lymph nodes and ankles of arthritic Rag2-KO mice in **E**. Compiled data from 2 independent experiments are shown in **E** and **F**. Each symbol in **A**–**C**, **E**, and **F** represent an individual mouse. Arthritis severity was quantified using the area under the curve. Graphs show mean ± SEM. *P < 0.05, **P < 0.01, ***P < 0.001 by Mann-Whitney *U* test (**E**), unpaired *t* test (**A**, **C**, and **F**) or 1-way ANOVA (**B**).

The abovementioned and other published evidence (22) in SKG mice points to a relationship between intestinal and joint inflammation in SKG mice and to the potential circulation of gut-induced IL-17–producing T cells as a potential pathogenic mechanism in this model. However, the cause-effect direction of such a relationship and whether intestinal autoreactive T cells play an arthritogenic role in SKG mice have not been addressed yet. To begin addressing these gaps in knowledge, we first compared the intestinal architecture in prearthritic SKG mice versus age-matched BALB/c mice. Consistent with previous reports (52), we did not find signs of ileal or jejunal inflammation in these mice; however, around 50% of prearthritic SKG mice showed histologically evident immune cell infiltration in the colonic lamina propria (Figure 1A). Immunophenotyping of prearthritic SKG versus age-matched BALB/c colonic lamina propria showed increased numbers of lymphocytes (CD45^+^ cells, Supplemental Figure 1A; supplemental material available online with this article; https://doi.org/10.1172/jci.insight.141868DS1). While a slight reduction was found in the numbers of CD4^+^ T cells (Supplemental Figure 1A), numbers of colonic RORγt^+^FoxP3^–^CD4^+^ T cells (CD45^+^TCRβ^+^CD4^+^RORγt^+^FoxP3^–^) and colonic Tregs (cTregs, CD45^+^TCRβ^+^CD4^+^FoxP3^+^) were significantly increased in prearthritic SKG mice versus BALB/c (Supplemental Figure 1A). We also observed a significant increase of RORγt-expressing (CD45^+^TCRβ^+^CD4^+^RORγt^+^FoxP3^+^) cTregs in SKG mice versus BALB/c (Figure 1A).

In line with reports that in other mouse strains RORγt-expressing Tregs mainly reside in the colons of healthy mice (53–55), we observed that RORγt-expressing Tregs were almost uniquely present in the colons of prearthritic SKG mice when compared with spleen and peripheral lymph nodes (Figure 1B and Supplemental Figure 1B). Expression of RORγt in CD4^+^ T cells, including Tregs, is highly dependent on IL-6 in B6 mice (55–57). Consistent with this notion, in prearthritic conditions, we found that IL-6–knockout (KO) SKG mice had a critical deficit of colonic RORγt-expressing Tregs and RORγt^+^FoxP3^–^CD4^+^ T cells, whereas the pool of total Tregs was unchanged in these mice (Figure 1C and Supplemental Figure 1C).

To assess whether the colonic lamina propria of SKG mice harbors arthritogenic T cells, we next isolated CD4^+^ T cells from either the spleen or the colonic lamina propria of prearthritic SKG mice, transferred them into recombination activating gene 2–KO (Rag2-KO) mice, and evaluated arthritis development in recipient mice (Figure 1D). As shown in Figure 1E, we found that colonic CD4^+^ T cells from prearthritic SKG mice induced significantly worse arthritis when compared with splenic CD4^+^ T cells from the same mice. Notably, there was a significantly increased accumulation of RORγt-expressing Tregs in addition to RORγt^+^FoxP3^–^CD4^+^ T cells in joint draining lymph nodes and ankles and of total Tregs in the ankles of mice that received colonic T cells (Figure 1F and Supplemental Figure 2).

Together, these results suggest that the colon of SKG mice is enriched with arthritogenic CD4^+^ T cells. Arthritis induced by colonic SKG T cell transfer is characterized by lymph nodes’ and ankles’ enrichment with RORγt-expressing T cells, a population that in healthy mice is highly enriched in the colon and whose generation is dependent on IL-6.

### Subclinical colonic inflammation promotes generation of arthritogenic CD4^+^ T cells and arthritis development in SKG mice.

**Figure 2:**
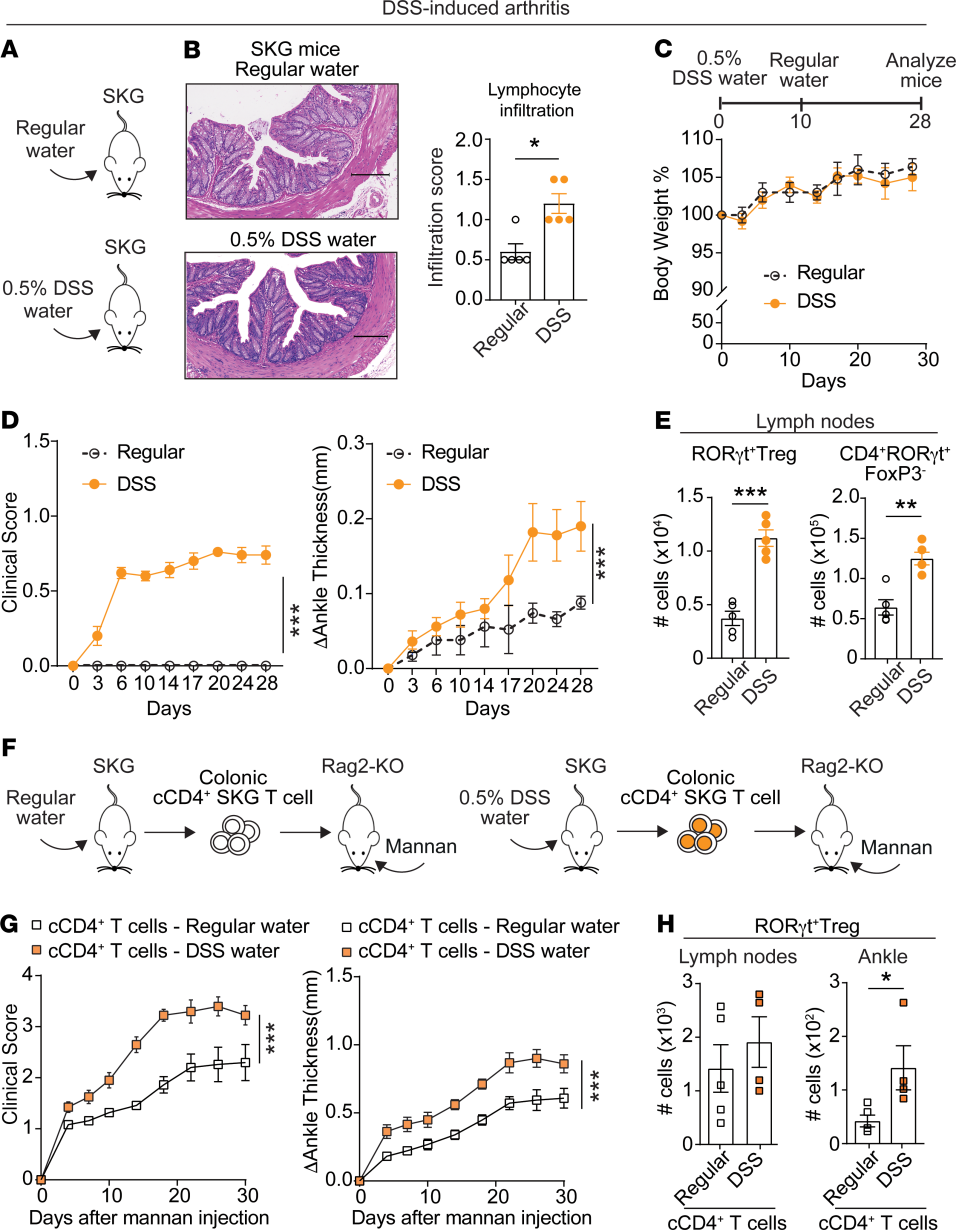
Colonic CD4^+^ T cells promote susceptibility to DSS-induced arthritis in SKG mice. (**A**) Female SKG mice received 0.5% DSS in their drinking water or regular drinking water (*n* = 5 mice/group) for 10 days, after which the water was replaced with regular drinking water until the end of the experiment. Arthritis was assessed by clinical scoring and measuring ankle thickness. Body weight was assessed twice per week. (**B**) *Left*: representative colon H&E staining. *Right:* colon lymphocyte infiltration score. Scale bars: 200 μm. (**C**) Change in body weight of mice in **A**. (**D**) Clinical score and change in ankle thickness of mice in **A**. (**E**) Numbers of RORγt^+^ Tregs (CD4^+^FoxP3^+^RORγt^+^) and RORγt^+^FoxP3^–^CD4^+^ T cells in lymph nodes of mice in **A**. (**F**) Adoptive transfer of colonic CD4^+^ T cells (cCD4^+^) isolated from mice receiving either 0.5% DSS water or regular water for 10 days into Rag2-KO recipient mice. Arthritis was induced 1 week after cell transfer by an intraperitoneal injection of mannan. (**G**) Clinical score and change in ankle thickness of mice in **F** receiving cCD4^+^ T cells from SKG mice treated with either 0.5% DSS water (*n* = 4) or regular water (*n* = 5). (**H**) Flow cytometric analysis of RORγt^+^ Tregs (CD4^+^FoxP3^+^RORγt^+^) cells in lymph nodes and ankles of arthritic Rag2-KO mice in **G**. Compiled data from 2 independent experiments are shown in **A**–**E**. Each symbol in **B**, **E**, and **H** represents an individual mouse. Arthritis severity was quantified using the area under the curve. Graphs show mean ± SEM. *P < 0.05, **P < 0.01, ***P < 0.001 by Mann-Whitney *U* test (**D** and **G**) or unpaired *t* test (**B**, **E**, and **H**).

Next, to investigate whether colonic inflammation can influence the severity of arthritis in SKG mice, we took advantage of dextran sulfate sodium (DSS) to disrupt colonic integrity (58). SKG mice were administered drinking water containing a low dose of DSS (0.5%), which was sufficient to enhance colonic inflammation (Figure 2, A and B) but did not induce clinically evident colitis (Figure 2C), or normal drinking water for 10 days and then maintained on normal drinking water for up to 28 days. Induction of subclinical colonic inflammation by 0.5% DSS led to development of clinically evident arthritis in SKG mice already at 28 days after DSS water, at an age when spontaneous arthritis was barely detectable by caliper measurement in our SKG colony (Figure 2D). DSS-induced arthritis in SKG mice was associated with significantly increased numbers of RORγt^+^FoxP3^–^CD4^+^ T cells, RORγt-expressing Tregs, as well as total Tregs in popliteal lymph nodes (Figure 2E and Supplemental Figure 3A).

To establish whether colonic inflammation enhances generation of arthritogenic CD4^+^ T cells, we isolated colonic CD4^+^ T cells from SKG mice administered either regular water or 0.5% DSS water for 10 days and transferred these to Rag2-KO mice (Figure 2F). As shown in Figure 2G, colonic CD4^+^ T cells from mice treated with DSS water induced significantly worse arthritis when compared with colonic CD4^+^ T cells from mice receiving regular water. This enhanced arthritis was associated with significant accumulation of RORγt^+^ Tregs and RORγt^+^FoxP3^–^CD4^+^ T cells in arthritic ankles, whereas no difference was observed in lymph nodes (Figure 2H and Supplemental Figure 3B).

These data suggest that subclinical colonic inflammation exacerbates arthritis in SKG mice by promoting expansion of arthritogenic colonic CD4^+^ T cells. In addition, arthritis induced by subclinical colon inflammation and transferred by colonic T cells in SKG mice is characterized by increased numbers of RORγt-expressing T cells, pointing to a potential colonic origin of these cells.

### Ptpn2 haploinsufficiency enhances SKG arthritis induced by subclinical colonic inflammation.

**Figure 3:**
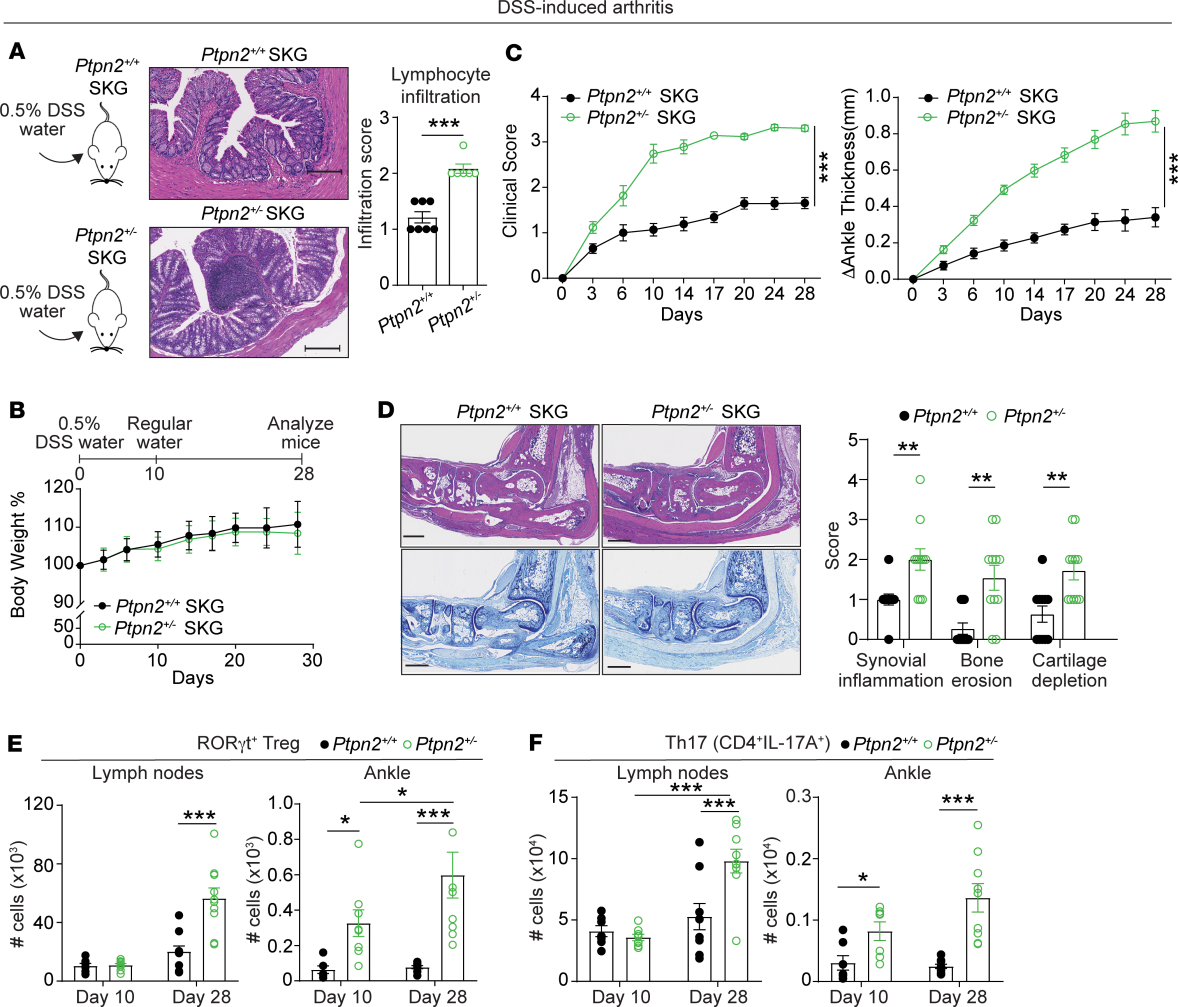
*Ptpn2* haploinsufficiency enhances susceptibility to DSS-induced arthritis in SKG mice. (**A**) *Left:* Female *Ptpn2^+/+^* (*n* = 7) and *Ptpn2^+/–^* (*n* = 6) SKG mice received 0.5% DSS in their drinking water for 10 days, after which the water was replaced with regular drinking water until the end of the experiment. *Middle:* representative colon H&E staining. *Right*: colon lymphocyte infiltration score. Scale bars: 200 μm. (**B**) Change in body weight of *Ptpn2^+/+^* (*n* = 13) and *Ptpn2^+/–^* (*n* = 12) SKG mice receiving 0.5% DSS in their drinking water as described in **A**. Body weight was measured twice per week. (**C**) Clinical score and change in ankle thickness of *Ptpn2^+/+^* (*n* = 13) and *Ptpn2^+/–^* (*n* = 12) SKG mice receiving 0.5% DSS in their drinking water as described in **A**. (**D**) *Left*: representative H&E and toluidine blue staining of arthritic ankles. *Right*: scores of synovial inflammation, cartilage depletion, and bone erosions. Scale bars: 500 μm. (**E** and **F**) Female *Ptpn2^+/+^* and *Ptpn2^+/–^* SKG mice received 0.5% DSS drinking water as in **A** and were sacrificed at 10 days (*n* = 7 *Ptpn2^+/+^* and *n* = 8 *Ptpn2*^+/–^ mice) or 28 days (*n* = 10 *Ptpn2^+/+^* and *n* = 10 *Ptpn2*^+/–^ mice). Number of (**E**) RORγt^+^ Tregs (CD4^+^FoxP3^+^RORγt^+^) and (**F**) Th17 (CD4^+^IL-17A^+^FoxP3^–^) in lymph nodes and ankles. Compiled data from 6 (**A**–**F**) independent experiments are shown. Each symbol in **A** and **D**–**F** represents an individual mouse. Arthritis severity was quantified using the area under the curve. Graphs show mean ± SEM. *P < 0.05, **P < 0.01, ***P < 0.001 by Mann-Whitney *U* test (**C**) or unpaired *t* test (**A** and **D**) or 2-way ANOVA (**E** and **F**).

We recently reported that *Ptpn2* haploinsufficiency in SKG mice enhances the development of mannan-induced arthritis and causes increased accumulation of RORγt-expressing Tregs in arthritic ankles (44) (Supplemental Figure 4). We thus sought to explore whether reduced PTPN2 expression would exacerbate the arthritogenicity of subclinical colonic inflammation in SKG mice. We administered 0.5% DSS to female *Ptpn2*-haploinsufficient and WT SKG mice to induce subclinical colonic inflammation as in Figure 2, A–C. This dose of DSS did not cause development of clinical colitis in mice of either genotype but resulted in more significant inflammatory infiltration in colons of *Ptpn2*^+/–^ mice when compared with WT mice (Figure 3, A and B). Increased colonic inflammation induced by 0.5% DSS correlated with dramatically enhanced arthritis development in *Ptpn2*-haploinsufficient SKG mice (Figure 3C), with increased signs of synovial inflammation, bone erosion, and cartilage depletion at 28 days after DSS water (Figure 3D). Consistent with the phenotype observed in *Ptpn2*^+/–^ SKG mice subjected to mannan-induced arthritis (44), numbers of Th17 (CD4^+^IL-17A^+^FoxP3^–^), total Tregs, and RORγt-expressing Tregs were increased in arthritic ankles and lymph nodes of mice with established (28 days) compared with early (10 days) arthritis. Th17 cells, total Tregs, and RORγt-expressing Tregs were also more expanded in the joints and lymph nodes of *Ptpn2*-haploinsufficient SKG mice with established arthritis. *Ptpn2*-haploinsufficient mice with early arthritis displayed a significant expansion of Th17 cells and RORγt-expressing Tregs only in arthritic ankles but not lymph nodes (Figure 3, E and F; and Supplemental Figure 5, A–D). Of notice, numbers of total T cells (TCRβ^+^ cells) and CD4^+^ T cells and of total cTregs and RORγt-expressing cTregs — but not of CD4^+^IL-17^+^FoxP3^–^ (cTh17) cells — were expanded in the colons of mice with established versus early DSS-induced arthritis (Supplemental Figure 5, E–H). However, when the colonic immunophenotype of *Ptpn2*-haploinsufficient mice was compared with WT mice, the only significant difference was a mild increase in total and CD4^+^ T cells in *Ptpn2*^+/–^ mice with established DSS-induced arthritis (Supplemental Figure 5, E and F). There was no other difference between *Ptpn2*^+/–^ and WT mice in total and CD4^+^ T cells, cTh17 cells, total cTregs, or RORγt-expressing cTregs in early or late DSS-induced arthritis (Supplemental Figure 5, E–H). Although we cannot rule out that additional differences might occur at earlier times, this colonic CD4^+^ T cell phenotype was consistent with the one observed in prearthritic SKG mice and after induction of mannan-induced SKG arthritis (Supplemental Figure 4E).

Induction of subclinical colonic inflammation in male *Ptpn2*-haploinsufficient SKG mice resulted in increased disease development and joint, lymph node, and colonic immunophenotypes similar to those observed in female mice (Supplemental Figure 6). DSS-induced arthritis was overall milder in male mice, consistent with previous observations that male SKG mice develop less severe arthritis (23) and that *Ptpn2* haploinsufficiency enhances arthritis less efficiently in male mice (44) (Supplemental Figure 6).

We conclude that *Ptpn2* haploinsufficiency enhanced disease severity and caused identical immunophenotypes — accumulation of IL-17^+^CD4^+^ T cells and colon-enriched RORγt-expressing Tregs in arthritic ankles and lymph nodes but not in the colonic lamina propria — in mannan-induced SKG arthritis (Supplemental Figure 4) and DSS-induced arthritis (Figure 3 and Supplemental Figures 5 and 6). Overall, the data suggest that *Ptpn2* haploinsufficiency links colonic inflammation to arthritis triggering or severity via local induction of arthritogenic colonic T cells or peripheral expansion of such cells once they have circulated to arthritic joints.

### PTPN2 maintains the stability of colonic Tregs in prearthritic SKG mice.

**Figure 4:**
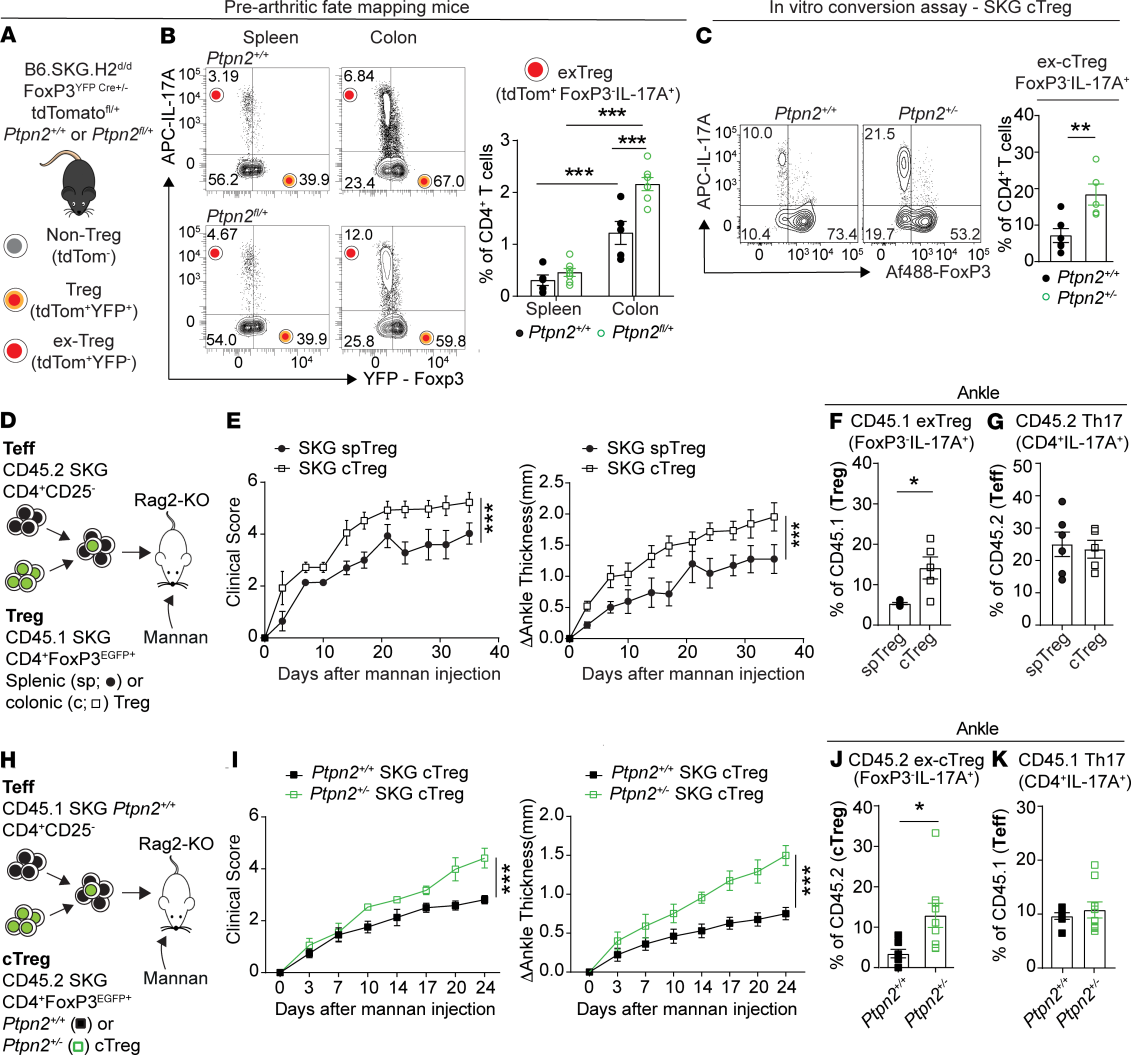
PTPN2 maintains the stability of colonic Tregs. (**A**) Schematic of SKG Treg fate-mapping mouse. (**B**) *Left:* representative flow cytometric gating of IL-17–producing cells among tdTomato^+^CD4^+^ T cells isolated from B6.SKG.H2^d/d^ FoxP3^YFP.Cre+/–^ tdTomato^fl/+^ *Ptpn2*^+/+^ or *Ptpn2*^fl/+^ Treg fate-mapping mice. *Right:* frequency of IL-17–producing exTregs (CD4^+^tdTomato^+^IL-17A^+^FoxP3^YFP–^) in spleens and colons isolated from 12-week-old prearthritic Treg fate-mapping SKG mice. (**C**) In vitro conversion assay of *Ptpn2^+/+^* (*n* = 6) and *Ptpn2^+/–^* (*n* = 5) colonic SKG Tregs (cTregs) into IL-17–producing ex-cTregs (CD4^+^IL-17^+^FoxP3^–^). Cells were analyzed after 4 days of stimulation. *Left:* representative flow cytometry plots. *Right:* frequency of ex-cTregs. (**D**–**G**) CD45.1 SKG CD4^+^ FoxP3^EGFP+^ colonic Tregs (cTregs) or splenic Tregs (spTreg) were cotransferred with CD45.2 SKG CD4^+^CD25^–^ effector T cells (Teffs) into Rag2-KO recipient mice (*n* = 4/group). Arthritis was induced in recipient mice 1 week after transfer by injection of mannan, and mice were analyzed at day 35. (**D**) Schematic for adoptive transfer experiment. (**E**) Clinical score and change in ankle thickness of recipient mice. (**F**) Frequency of CD45.1 IL-17A^+^ exTregs (CD4^+^IL-17^+^FoxP3^–^) in ankles of arthritic recipient mice. (**G**) Frequency of Th17 (CD4^+^IL-17A^+^FoxP3^–^) among transferred CD45.2 SKG CD4^+^CD25^–^ Teffs in ankles of arthritic recipient mice. (**H**–**K**) Cotransfer of CD45.2 SKG CD4^+^FoxP3^EGFP+^ *Ptpn2^+/+^* or *Ptpn2^+/–^* cTregs together with CD45.1 SKG CD4^+^CD25^–^ *Ptpn2^+/+^* Teff into Rag2-KO recipient mice (*n* = 7/group). Arthritis was induced in recipient mice 1 week after transfer by injection of mannan, and mice were analyzed at day 28. (**H**) Schematic for adoptive transfer experiment. (**I**) Clinical score and change in ankle thickness in recipient mice. (**J**) Frequency of *Ptpn2^+/+^* and *Ptpn2^+/–^* CD45.2 IL-17A^+^ ex-cTregs (CD4^+^IL-17A^+^FoxP3^–^) in ankles of arthritic recipient mice. (**K**) Frequency of Th17 (CD45.1^+^CD4^+^IL-17A^+^FoxP3^–^) among CD45.1 Teff in ankles of arthritic recipient mice. Compiled data from 2 independent experiments are shown in **A**–**K**. Each symbol in **B**, **C**, **F**, **G**, **J**, and **K** represents an individual mouse. Arthritis severity was quantified using the area under the curve. Graphs show mean ± SEM. *P < 0.05, **P < 0.01, ***P < 0.001 by 2-way ANOVA (**B**), unpaired *t* test (**C**, **F**, **G**, and **J**–**K**), or Mann-Whitney *U* test (**E** and **I**).

We have recently described a role for PTPN2 in inhibiting IL-6–dependent conversion of effector peripheral Tregs from SKG mice into arthritogenic IL-17–producing exTregs (44), by limiting IL-6–mediated downregulation of FoxP3. Thus, we next assessed whether *Ptpn2* haploinsufficiency enhances cTregs’ conversion in prearthritic SKG mice, by using our previously described “constitutive” Treg fate-mapping mice (44). These reporter mice (B6.SKG.H2^d/d^ FoxP3^YFP–Cre+/–^ tdTomato^fl/+^ *Ptpn2*^fl/+^) carry a constitutive Treg-specific haploinsufficiency of *Ptpn2*, a yellow fluorescence protein reporter that identifies cells currently expressing FoxP3 (Tregs) and a Cre-induced tdTomato reporter expressed from the Gt(ROSA)26 locus that identifies cells that are currently expressing (Tregs) or previously expressed (exTregs) FoxP3 (Figure 4A). Using these mice, we compared the presence of IL-17^+^ exTregs in the spleens and colons of SKG mice at the prearthritic stage. As shown in Figure 4B, IL-17^+^ exTregs were significantly increased in the colons versus spleens of prearthritic mice regardless of mouse genotypes. IL-17^+^ exTregs were also significantly increased in the colons but not the spleens of prearthritic mice carrying Treg-specific *Ptpn2* haploinsufficiency compared with WT mice (Figure 4B and Supplemental Figure 7). Consistent with the phenotype observed in the fate-mapping mouse, *Ptpn2* haploinsufficiency also markedly enhanced the conversion of cTregs from SKG mice into IL-17A^+^ exTregs (Figure 4C) in an in vitro Treg conversion assay (44).

The data suggest that in SKG mice the colon is a main site for the conversion of cTregs into IL-17^+^ exTregs already in the prearthritic state. This process is significantly enhanced by cell-intrinsic partial loss of PTPN2.

### PTPN2 maintains the stability of cTregs during autoimmune inflammation.

We next assessed whether cTregs’ instability contributes to the arthritogenic action of *Ptpn2*-haploinsufficient SKG colonic T cells. We adoptively transferred into Rag2-KO mice CD45.1-marked EGFP^+^ Tregs (CD4^+^FoxP3^EGFP+^) isolated from either the spleens or colons of FoxP3^EGFP^ SKG mice together with CD45.2-marked SKG CD4^+^ effector (CD4^+^CD25^–^) peripheral T cells and monitored development of arthritis after injection of mannan (Figure 4D and Supplemental Figure 8A). Notably, mice receiving cTregs developed significantly worse arthritis when compared with mice receiving splenic Tregs (Figure 4E). We observed an increased frequency of CD45.1^+^IL-17^+^ exTregs (CD4^+^IL-17A^+^FoxP3^EGFP–^) in both lymph nodes and ankles of mice receiving cTregs versus splenic Tregs (Figure 4F and Supplemental Figure 8B). However, no differences in the frequency of CD45.2^+^ Th17 (CD4^+^IL-17A^+^FoxP3^–^) cells were observed between the 2 groups of mice (Figure 4G and Supplemental Figure 8C), pointing to increased arthritis in recipients of cTregs being dependent on enhanced instability rather than reduced suppressive ability of cTregs.

Next, we cotransferred cTregs from either *Ptpn2*-haploinsufficient or WT CD45.2-marked FoxP3^EGFP^ SKG mice in combination with CD4^+^ effector T cells isolated from CD45.1-marked WT SKG mice and evaluated the development of arthritis after mannan injection (Figure 4H). As shown in Figure 4I, mice receiving *Ptpn2*-haploinsufficient cTregs developed significantly more arthritis when compared with mice transferred with WT cTregs. Flow cytometric analysis of arthritic ankles revealed an increased frequency of CD45.2^+^IL-17A^+^ exTregs in mice receiving *Ptpn2*-haploinsufficient cTregs (Figure 4J). A similar increase was observed in the lymph nodes of these mice (Supplemental Figure 8D). However, there was no difference in the frequency of CD45.1^+^ Th17 cells between mice transferred with either WT or *Ptpn2*-haploinsufficient cTregs, suggesting enhanced instability rather than reduced suppressive ability of *Ptpn2*-haploinsufficient cTregs (Figure 4K and Supplemental Figure 8E).

Together, these results suggest that cTregs could be an important source of pathogenic IL-17^+^ exTregs in arthritic SKG mice and that cTreg destabilization can mediate enhanced arthritis development in *Ptpn2*-haploinsufficient SKG mice.

### Arthritis in Ptpn2-haploinsufficient SKG mice is associated with accumulation of G protein–coupled receptor 15–positive CD4^+^ T cells.

**Figure 5:**
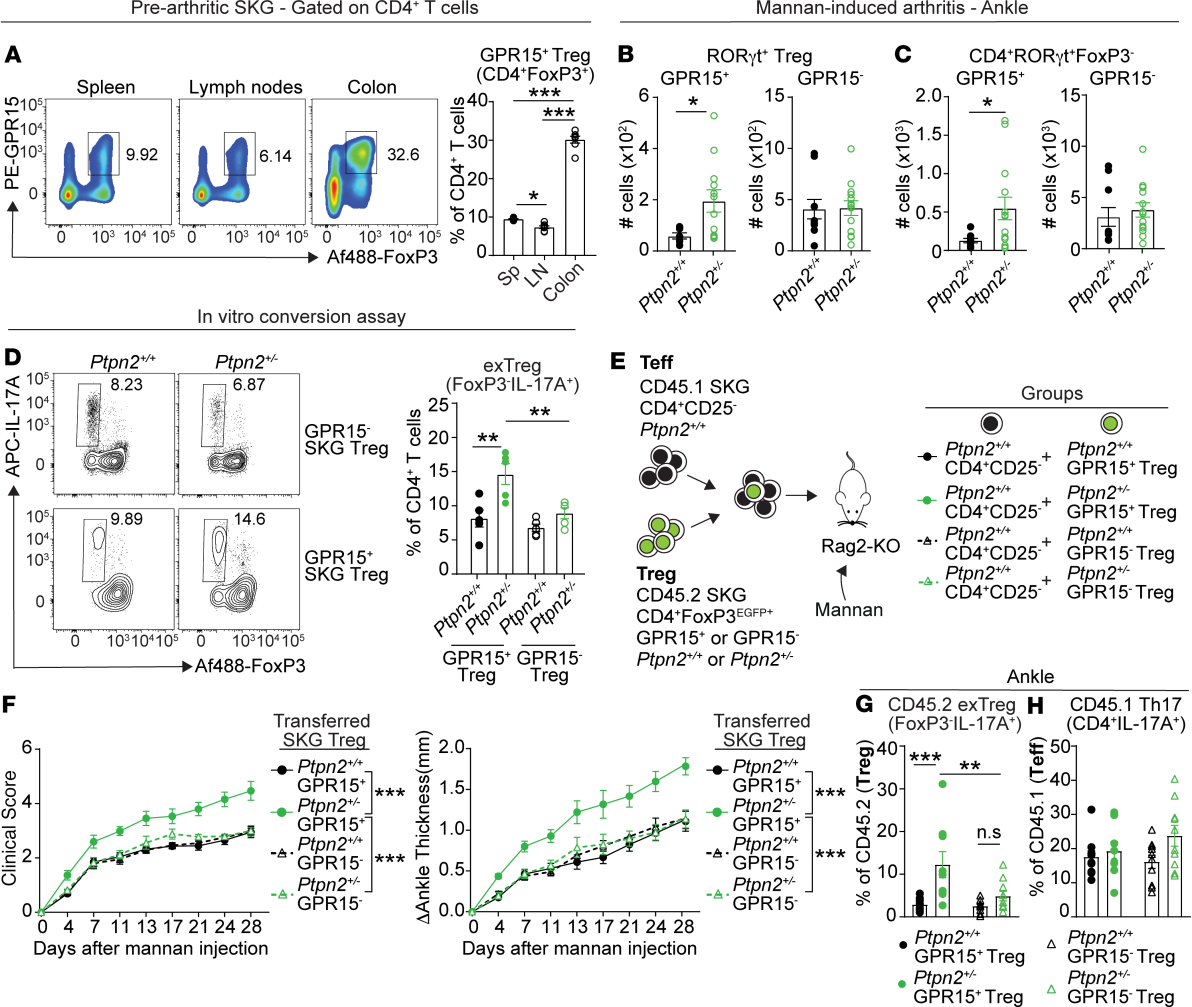
*Ptpn2* haploinsufficiency causes enhanced conversion of GPR15^+^ Tregs in vitro and in vivo. (**A**) Frequency of GPR15-expressing Tregs (CD4^+^FoxP3^+^GPR15^+^) among CD4^+^ T cells in spleen (Sp), lymph nodes (LN) and colons of prearthritic SKG mice (*n* = 7). (**B** and **C**) Number of GPR15^+^ and GPR15^–^ RORγt^+^ Tregs (CD4^+^RORγt^+^FoxP3^+^ GPR15^+^ or GPR15^–^) and RORγt^+^FoxP3^–^CD4^+^ T cells (CD4^+^RORγt^+^FoxP3^–^ GPR15^+^ or GPR15^–^) in ankles of *Ptpn2^+/+^* (*n* = 8) and *Ptpn2^+/–^* (*n* = 12) SKG mice with mannan-induced arthritis at day 49 shown in Supplemental Figure 4. (**D**) Frequency of IL-17A–producing exTregs (CD4^+^IL-17A^+^FoxP3^–^) after in vitro conversion of GPR15^+^ and GPR15^–^ Tregs isolated from *Ptpn2^+/+^* (*n* = 5) and *Ptpn2^+/–^* (*n* = 5) FoxP3^EGFP+^ SKG mice. (**E**–**G**) Cotransfer of CD45.1 SKG CD4^+^CD25^–^ *Ptpn2^+/+^* Teffs together with GPR15^+^ *Ptpn2^+/+^* (*n* = 10) or *Ptpn2^+/–^* (*n* = 9) Tregs (CD4^+^FoxP3^EGFP+^GPR15^+^) or GPR15^–^ *Ptpn2^+/+^* (*n* = 10) or *Ptpn2^+/–^* (*n* = 10) Tregs (CD4^+^FoxP3^EGFP+^GPR15^–^) into Rag2-KO mice. Arthritis was induced 1 week after cell transfer by an intraperitoneal injection of mannan. (**E**) Schematic for adoptive transfer experiment. (**F**) Clinical score and change in ankle swelling in Rag2-KO recipient mice. (**G**) Frequency of IL-17–producing exTregs (CD45.2^+^CD4^+^IL-17A^+^Foxp3^–^) in lymph nodes and ankles of arthritic recipient mice on day 28. (**H**) Frequency of Th17 (CD45.1^+^CD4^+^IL-17A^+^FoxP3^–^) among CD45.1 Teffs in lymph nodes and ankles of arthritic recipient mice on day 28. Compiled data from at least 2 (**A**) or 3 (**D**–**H**) independent experiments are shown. Each symbol in **A**–**D**, **G**, and **H** represents an individual mouse. Graphs show mean ± SEM.*P < 0.05, **P < 0.01, ***P < 0.001 by 1-way ANOVA (**A**), unpaired *t* test (**B** and **C**), Mann-Whitney *U* test (**F**), or 2-way ANOVA (**D**, **G**, and **H**).

To further characterize the role of PTPN2 in the stability of resident cTregs and determine whether there is a relationship between destabilization of cTregs and enhanced arthritis in *Ptpn2*-haploinsufficient mice, we focused on Tregs expressing G protein–coupled receptor 15 (GPR15). GPR15 identifies resident cTregs in mice (59–61). An analysis of GPR15 expression on CD4^+^ T cells in spleens, peripheral lymph nodes, and colons of SKG mice showed that GPR15^+^CD4^+^ T cells were overwhelmingly enriched in the colon and that they were mostly FoxP3^+^ (Figure 5A and Supplemental Figure 9, A and B). We next evaluated the number of total GPR15^+^ Tregs, GPR15^+^RORγt^+^FoxP3^–^CD4^+^ T cells, and GPR15^+^ RORγt-expressing Tregs in WT versus *Ptpn2*-haploinsufficient SKG mice subjected to mannan-induced arthritis. During mannan-induced arthritis, there were increased numbers of both GPR15^+^ RORγt-expressing Tregs and RORγt^+^FoxP3^–^CD4^+^ T cells but not of total GPR15^+^ Tregs in both popliteal lymph nodes and ankles of *Ptpn2*-haploinsufficient SKG mice. Strikingly, numbers of GPR15^–^ RORγt-expressing Tregs and GPR15^–^RORγt^+^FoxP3^–^CD4^+^ T cells were unaffected by *Ptpn2* haploinsufficiency (Figure 5, B and C; and Supplemental Figure 9, C–E). This phenotype was not due to increased local proliferation of GPR15^+^ cells because there was no difference in the expression of Ki-67, a cell proliferation marker, in total lymph node total GPR15^+^ Tregs, GPR15^+^ RORγt-expressing Tregs, or GPR15^+^RORγt^+^FoxP3^–^CD4^+^ T cells between WT and *Ptpn2*-haploinsufficient SKG mice (Supplemental Figure 9F).

Together, the data support the idea that *Ptpn2* haploinsufficiency enhances conversion of colonic GPR15^+^ cTregs into RORγt-expressing Tregs and exTregs either in the colon or after their egress and recruitment to arthritic joints. However, enhanced colonic egress and/or joint recruitment of GPR15^+^ T cells was not ruled out and could also play a role.

### PTPN2 selectively promotes the stability of GPR15^+^ Tregs in vitro and in vivo.

The above results led us to question whether PTPN2 differentially regulates the stability of GPR15^+^ versus GPR15^–^ Tregs. Thus, we sorted GPR15^+^ and GPR15^–^ Tregs from pooled spleens and colons of either *Ptpn2*-haploinsufficient or WT FoxP3^EGFP^ SKG mice and subjected these cells to in vitro IL-6–induced conversion (44). Notably, *Ptpn2* haploinsufficiency only caused increased conversion of GPR15^+^ Tregs into IL-17A–producing exTregs (Figure 5D).

Next, we sought evidence that the selective effect of PTPN2 on the stability of GPR15^+^ Tregs occurs in vivo and mediates the effect of *Ptpn2* haploinsufficiency on SKG arthritis. We isolated GPR15^+^ and GPR15^–^ Tregs from either WT or *Ptpn2*-haploinsufficient CD45.2 FoxP3^EGFP^ SKG mice and cotransferred these with CD4^+^ effector T cells isolated from CD45.1 WT SKG mice into Rag2-KO mice, then monitored development of arthritis after injection of mannan (Figure 5E). Consistent with the abovementioned in vitro data, only mice receiving *Ptpn2*-haploinsufficient GPR15^+^ Tregs developed significantly more severe arthritis compared with the other groups (Figure 5F), whereas arthritis development in mice receiving *Ptpn2*-haploinsufficient GPR15^–^ Tregs was comparable to mice receiving either WT GPR15^+^ or GPR15^–^ Tregs (Figure 5F). Flow cytometric analysis of popliteal lymph nodes and ankles of arthritic mice revealed increased frequencies of CD45.2^+^ IL-17A–producing exTregs in mice receiving *Ptpn2*-haploinsufficient GPR15^+^ Tregs (Figure 5G and Supplemental Figure 10A), supporting a selective effect of *Ptpn2* haploinsufficiency on GPR15^+^ Treg stability. Furthermore, consistent with the arthritis phenotype, no differences in exTregs were observed between mice receiving either *Ptpn2*-haploinsufficient GPR15^–^ Tregs or WT GPR15^+^ Tregs or GPR15^–^ Tregs (Figure 5G and Supplemental Figure 10A). We did not observe any differences in the numbers of exTregs in the colon between all transfer groups (Supplemental Figure 10C). CD45.1^+^ Th17 cells derived from transferred CD4^+^ effector cells were also comparable between the groups (Figure 5H and Supplemental Figure 10, B and D). To confirm that the observed differential effect of *Ptpn2* haploinsufficiency was not due to selective alteration of the suppressive capacity of either GPR15^+^ or GPR15^–^ Tregs, we also performed in vitro Treg suppression assays (Supplemental Figure 10, E and F). Although *Ptpn2* haploinsufficiency did not alter the suppressive capacity of either GPR15^+^ or GPR15^–^ Tregs, GPR15^+^ Tregs showed increased suppressive capacity when compared with GPR15^–^ Tregs regardless of *Ptpn2* genotype (Supplemental Figure 10E).

Together, these results strongly suggest that increased arthritis development observed in mice receiving *Ptpn2*-haploinsufficient Tregs is due to selectively increased destabilization of GPR15^+^ Tregs.

### Treg-specific Ptpn2 haploinsufficiency enhances SKG arthritis induced by subclinical colonic inflammation.

**Figure 6:**
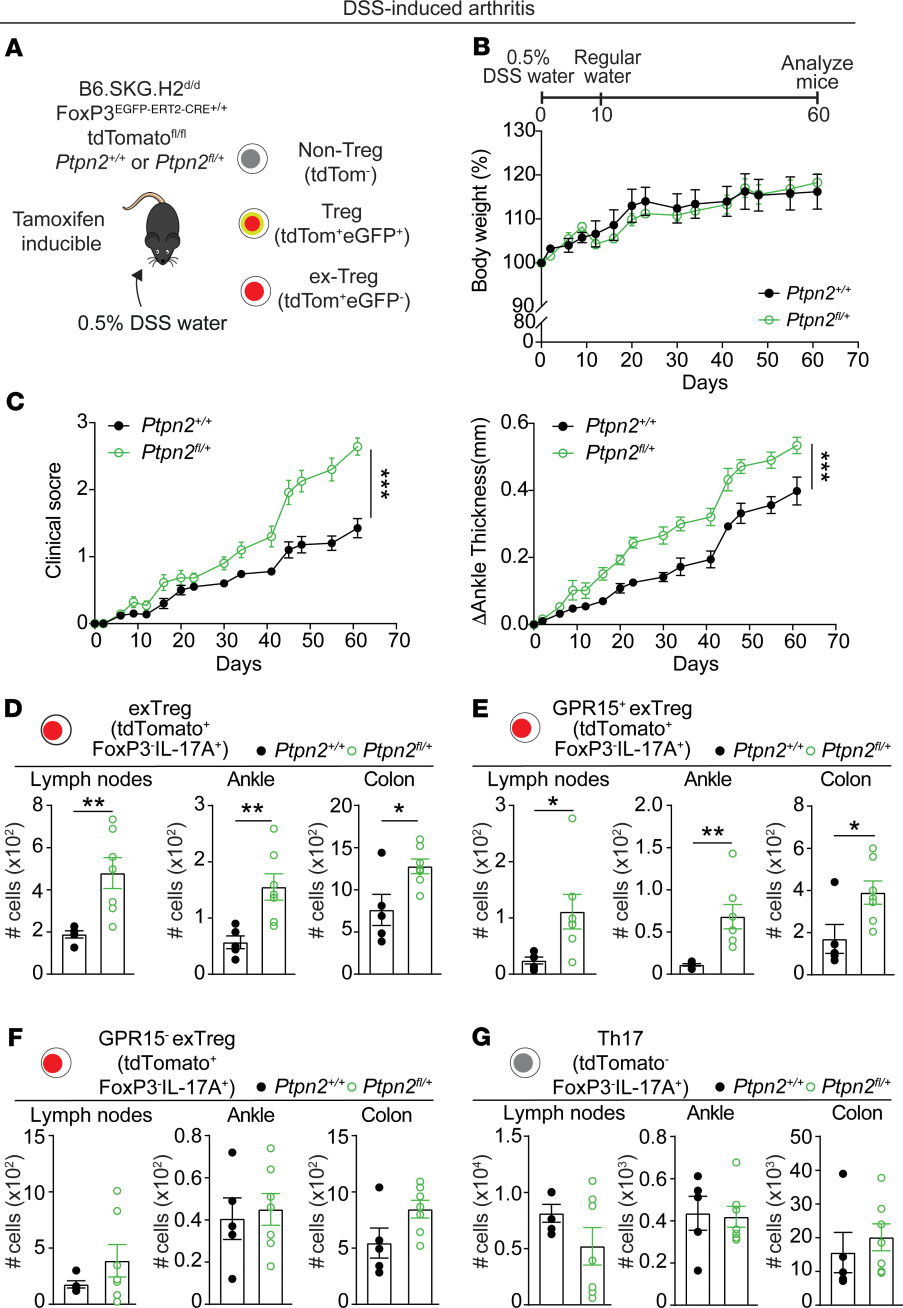
Inducible Treg-specific *Ptpn2* haploinsufficiency aggravates development of DSS-induced arthritis. (**A**) Schematic of B6.SKG.H2^d/d^ FoxP3^EGFP-ERT2-Cre+/+^ tdTomato^fl/fl^ *Ptpn2^+/+^* or *Ptpn2*^fl/+^ inducible Treg fate-mapping mice. (**B**–**G**) Female *Ptpn2^+/+^* (*n* = 5) and *Ptpn2*^fl/+^ (*n* = 7) inducible Treg fate-mapping mice received 0.5% DSS in their drinking water for 10 days. After 10 days, the water was replaced with regular drinking water until the end of the experiment at day 61. Treg fate-mapping mice were administered 100 μL tamoxifen (20 mg/mL) via oral gavage for 5 consecutive days to induce Cre expression. After the last treatment with tamoxifen, mice were rested for 2 weeks before receiving DSS drinking water. (**B**) Change in body weight. (**C**) Clinical score and change in ankle thickness. (**D**) Number of IL-17–producing exTregs (CD4^+^tdTomato^+^IL-17A^+^FoxP3^–^) in lymph nodes, ankles, and colons. (**E** and **F**) Number of IL-17–producing (**E**) GPR15^+^ or (**F**) GPR15^–^ exTregs (CD4^+^tdTomato^+^IL-17A^+^FoxP3^–^) in lymph nodes, ankles, and colons. (**G**) Number of tdTomato^–^ Th17 (CD4^+^tdTomato^–^IL-17A^+^FoxP3^–^) in lymph nodes, ankles, and colons. Compiled data from 2 independent experiments are shown. Clinical score and ankle swelling were quantified using the area under the curve. Each symbol in **D**–**G** represents an individual mouse. Graphs show mean ± SEM *P < 0.05, **P < 0.01, ***P < 0.001 by Mann-Whitney *U* test (**C**) or unpaired *t* test (**D**–**G**).

To gather further evidence that promotion of cTregs’ conversion into IL-17^+^ T cells contributes to the observed enhancement of DSS-induced SKG arthritis by *Ptpn2* haploinsufficiency, we bred *Ptpn2*^fl/+^ SKG mice with a tamoxifen-inducible Treg fate-mapping model (44). These reporter mice (B6.SKG.H2^d/d^ FoxP3^EGFP-ERT2Cre+/+^ tdTomato^fl/fl^ *Ptpn2*^fl/+^) carry tamoxifen-inducible Treg-specific haploinsufficiency of *Ptpn2* and EGFP expression and tdTomato reporter expression from the Gt(ROSA)26 locus that identifies cells that are currently expressing (Tregs) or at some point expressed FoxP3 (exTregs) (Figure 6A). Because tamoxifen treatment is performed only in the prearthritic and pre-DSS phase, cells undergoing induction of FoxP3 exclusively during the course of inflammation, such as the majority of locally induced Tregs in inflamed joints, are excluded from the tracking. Tamoxifen-induced *Ptpn2* haploinsufficiency in these mice enhanced mannan-induced arthritis similar to that previously reported in the constitutive fate-mapping *Ptpn2*-haploinsufficient mice (Supplemental Figure 11A) (44). We next assessed the effect of tamoxifen-inducible Treg-selective haploinsufficiency of *Ptpn2* in the context of DSS-induced arthritis. As shown in Figure 6, B and C, *Ptpn2* haploinsufficiency in tamoxifen-inducible fate-mapping mice led to enhanced DSS-induced arthritis. This phenotype correlated with an increased presence of GPR15^+^ IL-17–producing exTregs in joints, lymph nodes, and colons (Figure 6, D and E; and Supplemental Figure 11B), while differences in numbers of GPR15^–^ IL-17–producing exTregs were minimal and nonsignificant (Figure 6F). On the other hand, numbers of Th17 (tdTomato^–^CD4^+^IL-17A^+^FoxP3^–^) cells were similar between mice with Treg-selective haploinsufficiency of *Ptpn2* and WT mice (Figure 6G).

Overall, these results support the idea that *Ptpn2* haploinsufficiency enhances DSS-induced arthritis in SKG mice by inducing a Treg-intrinsic reduction in cTreg stability, which is mainly restricted to GPR15^+^ cells. The marked accumulation of GPR15^+^ exTregs in the joints and lymph nodes of *Ptpn2*^+/–^ fate-mapping mice after low-dose DSS treatment suggests that cTregs’ instability at least in part links subclinical colonic inflammation to enhanced arthritis in these mice.

## Discussion

**Figure 7:**
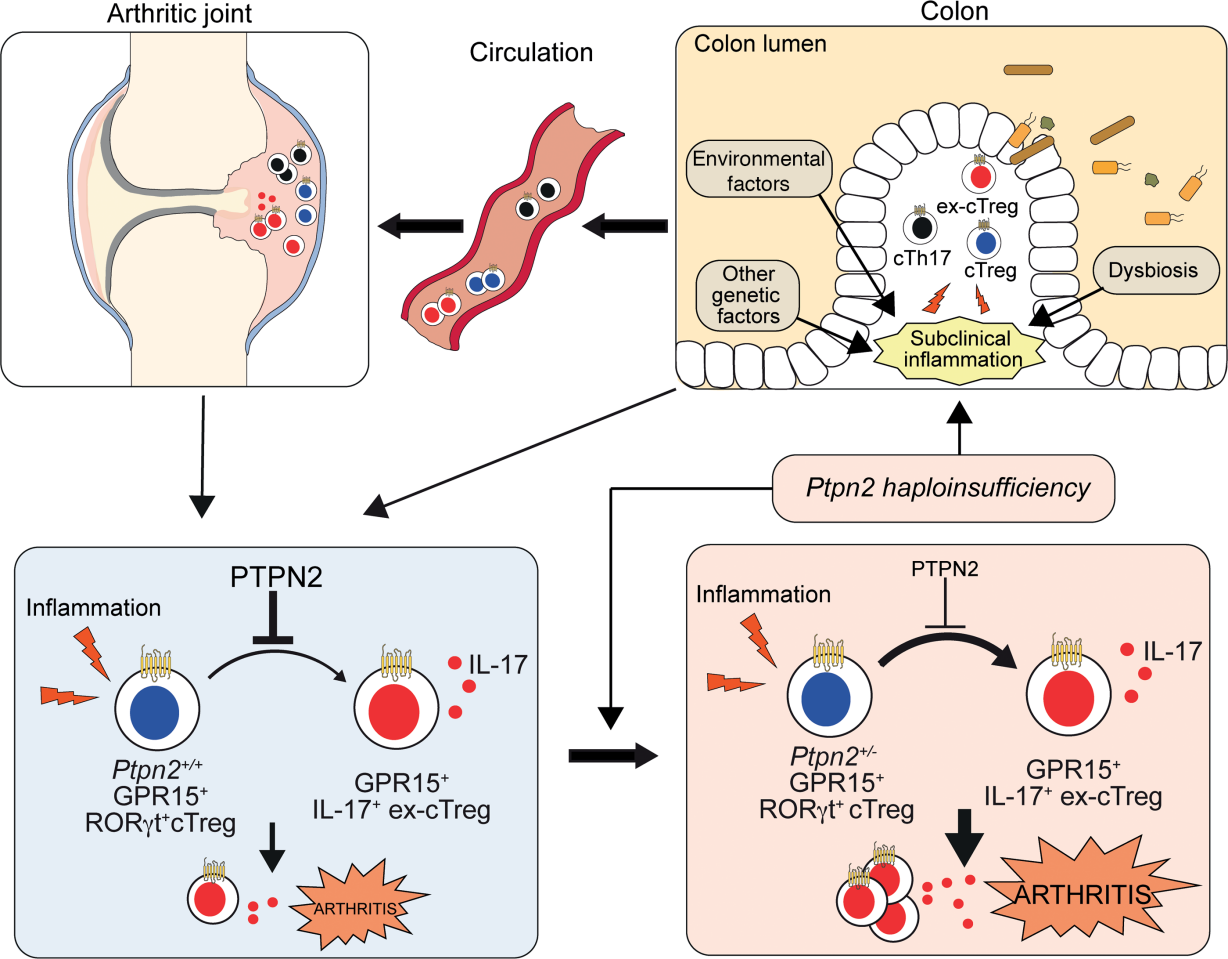
Proposed model of how PTPN2-controlled stability of GPR15-expressing colonic Tregs affects the gut-joint inflammation axis in autoimmune arthritis. Subclinical colonic inflammation promotes both expansion of autoreactive CD4^+^ T cells as well as instability of GPR15-expressing RORγt^+^ cTregs, resulting in egress of autoreactive GPR15-expressing RORγt^+^ cTregs, ex-cTregs, and cTh17 to the joints. Loss of PTPN2 impinges on this process via a Treg-intrinsic mechanism by further reducing cTreg stability at the colonic level or in arthritic joints or at both locations, which amplifies both colonic and subsequent joint inflammation.

Experimentation in mouse models other than SKG has shown that the gut can host arthritogenic T cells (18, 20). However, it remains unclear to which extent the relationship between gut inflammation and arthritis is due to an enrichment of arthritogenic T cells within the gut versus promotion of systemic T cell activation. Here, we established a cause-effect relationship between subclinical intestinal inflammation and enhanced arthritis severity in SKG mice, a model that is known to develop both intestinal inflammation and gut microbiome–dependent arthritis (21, 22, 52). We also leveraged the SKG model to demonstrate that partial loss of function of PTPN2, mimicking common genetic *PTPN2* variations associated with both RA and IBD (2, 25, 32–34), in Tregs, interacts with gut inflammation to promote severity of arthritis. In our current working model (shown in Figure 7), subclinical colonic inflammation promotes both expansion of autoreactive T cells in the colon as well as cTregs’ instability and egress of autoreactive cTregs — but also likely of RORγt-expressing Tregs, ex-cTregs, and cTh17 to the joints. Loss of PTPN2 impinges on this arthritogenic mechanism via a Treg-intrinsic mechanism by further reducing cTreg stability at the colonic level or in arthritic joints or at both locations. Loss of PTPN2 also enhances intestinal inflammation in SKG mice, potentially through the same effect on cTreg stability and/or additional effects of PTPN2 on lymphocyte and myeloid cells (32, 45, 46). Regulation of colonic T cell egress by PTPN2 via enhanced colonic inflammation or more direct effects is also likely and warrants further investigation.

The intestinal mucosa is enriched with Th17 cells, and previous studies have shown that colonic T cells can be arthritogenic (18, 53). GPR15 is important for the homing of Tregs to the colon and for the maintenance of colonic immune homeostasis in mice (59, 61, 62). GPR15 can therefore be used as a marker for murine colon-resident Tregs. Many of the cTregs show a Th17-like phenotype, expressing the transcription factor RORγt, and the generation of these RORγt-expressing Tregs is at least partially IL-6 dependent (55). These cells have an activated phenotype and play an important role in limiting Th17-mediated immune responses not only in the gut but also in the periphery during autoimmune inflammation (54, 55, 63). In line with this notion, we find RORγt-expressing Tregs mainly within the colons of prearthritic SKG mice, where their generation is dependent on IL-6. During SKG mannan-induced and DSS-induced arthritis, there is an expansion of RORγt-expressing Tregs in joint draining lymph nodes and arthritic ankles. It is likely that this expansion is in part due to local inflammation-induced RORγt upregulation, but further experimentation is necessary in order to distinguish RORγt-expressing Tregs that are joint recruited from those that are induced locally in the context of arthritic inflammation. Nevertheless, joint and joint draining lymph node enrichment of GPR15^+^ total Tregs and GPR15^+^ RORγt-expressing Tregs observed in SKG mice with Treg-specific haploinsufficiency of *Ptpn2* induced in concomitance with DSS treatment supports the idea of an early conversion of autoreactive cTregs or their egress to the joints. The earlier appearance of GPR15^+^ RORγt-expressing Tregs in ankles versus lymph nodes is also consistent with migration of circulating cTregs into inflamed joints, where it has been reported that Tregs’ conversion can be driven by synoviocyte-produced IL-6 (51).

Compared with peripheral Tregs, SKG cTregs have an increased tendency to lose FoxP3 expression and convert into IL-17–expressing arthritogenic exTregs after adoptive transfer into RAG mice. This observation is consistent with our finding that effector Tregs have an increased sensitivity to IL-6–promoted conversion into pathogenic IL-17–producing exTregs, which correlates with their increased expression of RORγt (44, 54, 55). Intriguingly, we find that the effect of *Ptpn2* haploinsufficiency on Treg stability is limited to GPR15-expressing colonic Tregs. These cells also show an increased suppressive capacity compared with GPR15^–^ Tregs, which is unaffected by PTPN2 but might partially balance their instability. The latter observation might explain why there is no difference in arthritis development between Rag2-KO mice receiving GPR15^+^ (which are predominantly cTregs) versus GPR15^–^ WT Tregs.

DSS treatment induced an expansion of colonic Tregs and Th17 in SKG mice. However, the frequency of these T cell populations was not increased in DSS-treated versus prearthritic colons. There was also no differential expansion of these populations in the colons of *Ptpn2^+/+^* versus *Ptpn2*^+/–^ mice after DSS treatment, despite an increased infiltration of total immune cells and T cells in *Ptpn2*^+/–^ mice. The lack of correlation between joint and colonic expansion of Tregs and Th17 supports our hypothesis of an enhanced colonic egress of cTregs. However, our histopathological analysis of immune cell infiltration was limited to the distal part of the colon, whereas the flow cytometric analysis of CD4^+^ T cells was performed on whole colons at more than 10 days after DSS mark. Thus, our study might be insensitive to localized expansions of Tregs or Th17 cells or to potentially transient phenomena that occur before the 10-day after DSS mark.

In addition to the known caveats of modeling human arthritis in mice (19, 64), the direct translatability of our findings in SKG arthritis might be limited by differences in the expression patterns of GPR15 between human and mouse immune cells (in humans, GPR15 is mainly found on effector cells and to lesser extent on Tregs, refs. 61, 65). Further experimentation is also needed to understand the molecular basis of the selective effect of *Ptpn2* haploinsufficiency in GPR15^+^ versus GPR15^–^ Tregs and whether the effect of PTPN2 is limited to IL-6–induced Treg instability. Also, PTPN2 regulates epithelial gut barrier functions (66), and its complete loss can affect the gut microbiome in mice (46). Thus, it is possible that gut dysbiosis also contributes to enhancing arthritis severity in *Ptpn2*-haploinsufficient SKG mice.

Despite these limitations, our study does support the idea that autoimmune-associated haploinsufficiency in *Ptpn2* promotes development of arthritis through an action on a population of cTregs expressing GPR15. Such destabilization of cTregs might also play a role in the mechanism of action of *PTPN2* in IBD. No genetic association between *PTPN2* and SpAs has emerged so far; however, to the best of our knowledge, GWAS in SpAs have been limited to ankylosing spondylitis and psoriatic arthritis (3, 67). Thus, we also suggest that a potential role of *PTPN2* in enteropathic arthritis is worth exploration.

Our model contributes to clarifying how genetic risk factors for autoimmunity interact with the gut microenvironment in the pathogenesis of disease and is particularly fitting for genes, such as *PTPN2*, that are shared between IBD and RA or SpA. We also validated the SKG mouse as a model to study the gut-joint axis and established a potentially novel model of arthritis triggered by subclinical colonic inflammation. Considering that IL-17 is believed to play a more critical role in SpA versus RA (48, 68) and the emerging importance of SKG mice as a model for SpA inflammation, our colitis-induced model might also be applicable to studying the mechanism of action of SpA genes, and especially enteropathic arthritis.

## Methods

### Mice.

BALB/c SKG, BALB/c *Ptpn2*^+/–^, and C57BL/6 (B6) *Ptpn2*-floxed (*Ptpn2*^fl/fl^) mice have been previously described (37, 69, 70). B6 mice congenic for the H2^d^ haplotype (JAX 000359, B6.C-H2^d^ /bByJ), B6 FoxP3^YFP-Cre^ [JAX 016959, B6.129(Cg)-Foxp3^tm4(YFP/iCre)Ayr^/J (71)], FoxP3^EGFP-ERT-Cre^ [JAX 016961, B6.Foxp3tm9(EGFP/cre/ERT2)Ayr/J (72)], B6 ROSA-26-tdTomato [JAX 007914, B6;Cg-Gt(ROSA)26Sor^tm14(CAG-tdTomato)Hze^/J (73)], BALB/c FoxP3-EGFP [JAX 006769, C.Cg-Foxp3^tm2Tch^/J (74)], BALB/c CD45.1 [JAX 006584, CByJ.SJL(B6)-Ptprc^a^/J], BALB/c *Il6*^–*/*–^ [JAX 007078, CByJ.129S2(B6)-*Il6^tm1Kopf^*/J], and BALB/c (JAX 000651, BALB/cJ) mice were all purchased from The Jackson Laboratory. Breeding onto the SKG background was performed in-house. BALB/c Rag2-KO mice were purchased from Taconic (model 601). All mice were housed in the UCSD vivarium under specific pathogen–free conditions.

### Mannan-induced arthritis.

Male or female 8-week-old SKG mice were injected i.p. with 20 mg of mannan (M7504, MilliporeSigma), dissolved in sterile PBS. Clinical scoring and measurement of ankle thickness using a digital caliper were performed twice weekly according to an established protocol (23, 44). Clinical signs of arthritis in front and hind paws were scored as follows: 0, no joint swelling; 0.1 per swollen digit (3 digits on front paw and 4 digits on hind paw); 0.5, mild swelling of wrist or ankle; and 1.0, severe swelling of wrist or ankle. Scores for all digits of fore paws and hind paws, wrists, and ankles were combined for each mouse, yielding a maximum score of 5.4, which was considered the clinical endpoint. Mice reaching clinical endpoint scores were sacrificed according to ethical guidelines. All arthritis studies were performed on littermate mice. Clinical scoring of mice was performed in a blinded manner in which genotypes were unknown to the researcher during scoring.

### DSS-induced arthritis.

For induction of intestinal inflammation, drinking water containing 0.5% DSS (MP216011025, MP Biomedicals) was administered to 8-week-old female or male *Ptpn2^+/+^* or *Ptpn2*^+/–^ SKG mice for 10 days. After 10 days, the DSS-containing drinking water was replaced with regular drinking water until the end of the experiment. Body weight was monitored twice per week to determine potential development of colitis. Development of arthritis in littermate mice was evaluated in a blinded manner by clinical scoring and measurement of ankle thickness as described above.

### Constitutive and inducible Treg fate-mapping mice.

For evaluation of Treg fate in vivo, we used our recently described constitutive Treg fate-mapping mice (44) on the SKG background (B6.SKG.H2^d/d^ FoxP3^YFP-Cre+/–^ tdTom^fl/+^ *Ptpn2*^+/+^ or *Ptpn2*^fl/+^) and inducible Treg fate-mapping mice that were generated by crossing B6 FoxP3^EGFP-ERT2-Cre+/+^ mice with B6 ROSA-26-tdTomato^fl/fl^, B6 *Ptpn2*^fl/fl^, and B6.SKG.H2^d/d^ mice. In the resulting B6.SKG.H2^d/d^ FoxP3^EGFP-ERT2-Cre+/+^ tdTomato^fl/fl^ *Ptpn2^+/+^* or *Ptpn2*^fl/+^ mice, FoxP3^EGFP+^ Tregs carry an inducible Cre-ERT2 fusion protein that can be activated by administration of tamoxifen, allowing for generation of Treg-specific *Ptpn2* haploinsufficiency and fate mapping. To induce Cre expression, 8-week-old *Ptpn2^+/+^* and *Ptpn2*^fl/+^ female inducible Treg fate-mapping mice were administered 100 μL tamoxifen (T5648, MilliporeSigma, 20 mg/mL dissolved in corn oil) via oral gavage (for DSS treatment) or by i.p. injection (for mannan-induced arthritis) for 5 consecutive days (75). Two weeks after the last treatment, a blood sample was collected by retro-orbital bleeding, and tdTomato expression in EGFP^+^ (FoxP3^+^) CD4^+^ T cells was analyzed by flow cytometry to confirm successful induction of Cre expression by tamoxifen. Tamoxifen-treated mice were either treated with 0.5% DSS drinking water or administered a single i.p. injection of 20 mg of mannan to induce arthritis as described above.

### Histological assessment of arthritic joints.

Hind paws were fixed in 10% neutral-buffered zinc/formalin, decalcified, and embedded in paraffin. Sections were prepared from tissue blocks by HistoTox and stained with H&E and toluidine blue. Histopathological evaluation of synovial inflammation and bone erosion was performed from H&E-stained slides and cartilage erosion from toluidine blue–stained slides. Scoring of synovial inflammation, bone destruction, and cartilage depletion was performed in a blinded manner by 2 independent researchers as previously described (76).

### Histological assessment of colons.

The colon was separated into 3 parts: proximal, middle, and distal. After removing feces, each colon sample was fixed in 10% neutral-buffered formalin. The 3 parts of the colon were then embedded in a single paraffin block, sectioned, and stained with H&E. Histopathological scoring for infiltration was performed in a blinded manner as previously described (77). Histological signs of immune infiltration were scored as follows: 0, no infiltrate; 1, infiltrate around crypt basis; 2, infiltrate reaching to muscular mucosae; 3, extensive infiltration reaching the muscular mucosae and thickening of the mucosa with abundant edema; and 4, infiltration of the submucosa.

### Cell preparation and flow cytometry.

Single-cell suspensions were prepared from lymph nodes and spleens by mincing the tissues through a 70 μm cell strainer. Isolation of cells from the colonic lamina propria was performed according to a previously published protocol (78). Briefly, colons were segmented and washed with Hanks’ balanced salt solution (HBSS), after which the mucosal layer was removed using fetal bovine serum–free (FBS-free) HBSS containing 25 mM HEPES and 20 mM EDTA. The colonic tissue was then dissociated using collagenase VIII (C2139, MilliporeSigma, 0.67 mg/mL) and DNase I (10104159001, Roche, 0.05 mg/mL) for 30 minutes at 37°C, after which lymphocytes were isolated using a Percoll gradient. For isolation of synovial cells, joints were rinsed with 5% FBS RPMI medium and dissociated with collagenase VIII (0.67 mg/mL) and DNase I (0.05 mg/mL) at 37°C for 40 minutes. Cells were preincubated with Fc block (2.4G2, BD Pharmingen) before antibody staining. For surface staining, fluorescence-conjugated antibodies against CD4 (RM4-5), CD8 (53-6.7), TCRβ (H57-597), CD45.2 (30F11), CD45.1 (A20), and CD25 (PC65.1) were purchased from eBioscience, Thermo Fisher Scientific. The anti-GPR15 antibody (S150421) was obtained from BioLegend. For intracellular cytokine staining, cells were incubated with 20 ng/mL phorbol 12-myristate 13-acetate (PMA, P8139, MilliporeSigma) and 1 μM ionomycin (I0634, MilliporeSigma) in the presence of brefeldin A (1:1000 dilution, 00-4506-51, eBioscience, Thermo Fisher Scientific) for 5 hours at 37°C. Intracellular staining was performed with the IC fixation buffer (50-112-9058, eBioscience, Thermo Fisher Scientific) and permeabilization buffer (00-5123-43, eBioscience, Thermo Fisher Scientific). For intracellular staining of transcription factors, the FoxP3/Transcription Factor staining buffer set was used (50-112-8857, eBioscience, Thermo Fisher Scientific). Antibodies against FoxP3 (FJK-16s), RORγt (B2D), Ki-67 (SolA15), and IFN-γ (XMG1.2) were obtained from eBioscience, Thermo Fisher Scientific. The anti–IL-17A antibody (TC11-18H10.1) was purchased from BioLegend. Dead cells were excluded by staining with Fixable Viability dye (65-0866-18) from eBioscience, Thermo Fisher Scientific. Cells were analyzed on a Bio-Rad ZE5 Cell Analyzer. Flow-sorting was performed on a FACSARIA II (BD). Flow cytometry data were analyzed using FlowJo software (Tree Star, Inc.).

### CD4^+^ T cell transfer in Rag2-KO mice.

CD4^+^ SKG T cells (1 × 10^6^) flow-sorted from either the spleen or the colonic lamina propria of 10-week-old female CD45.1^+^ FoxP3^EGFP+^ SKG mice were transferred into 8-week-old female CD45.2 Rag2-KO BALB/c mice through retro-orbital injection. One week after transfer, arthritis was induced in recipient mice by a single i.p. injection with 20 mg mannan. Development of arthritis in littermate mice was evaluated in a blinded manner by clinical scoring and measurement of ankle thickness as described above. To evaluate the effect of subclinical colonic inflammation on the generation of arthritogenic CD4^+^ T cells, female SKG mice were administered either 0.5% DSS water or regular water as described above. At day 10, CD4^+^ T cells (1.5 × 10^5^) were flow-sorted from the colonic lamina propria and transferred into 8-week-old female BALB/c Rag2-KO mice through retro-orbital injection. Arthritis was induced and monitored as described above.

### In vitro Treg conversion assay.

In vitro conversion of FoxP3^+^ SKG Tregs was performed using our previously established protocol, which is adopted from a protocol by Komatsu et al. (44, 51). For experiments presented in Figure 4C, colonic CD4^+^FoxP3^EGFP+^ SKG Tregs were flow-sorted from 8- to 10-week-old male or female *Ptpn2^+/+^* and *Ptpn2^+/–^* FoxP3^EGFP^ SKG mice. For experiments in Figure 5D, GPR15^+^ or GPR15^–^ CD4^+^FoxP3^EGFP+^ SKG Tregs were flow-sorted from pooled spleens and colons isolated from either *Ptpn2^+/+^* or *Ptpn2^+/–^* 8- to 10-week-old male or female FoxP3^EGFP^ SKG mice. Sorted Tregs were stimulated with Dynabeads mouse anti-CD3/CD28 T cell activation beads (11452D, Invitrogen, Thermo Fisher Scientific) in the presence of IL-6 (575702, BioLegend; 50 ng/mL) for 96 hours. After 96 hours, cells were restimulated with PMA (20 ng/mL), ionomycin (1 μM), and brefeldin A for 5 hours and analyzed for the expression of IL-17A, FoxP3, and RORγt using flow cytometry.

### In vivo Treg stability assays in Rag2-KO mice.

For evaluation of Treg stability during arthritis development, we used our previously established protocol (44). For experiments evaluating the difference between colonic and peripheral Tregs (Figure 4, D–G; and Supplemental Figure 8), CD4^+^ FoxP3^EGFP+^ SKG Tregs were flow-sorted from spleen or the colonic lamina propria of 8- to 10-week-old female *Ptpn2^+/+^* CD45.1 FoxP3^EGFP^ SKG mice, whereas CD4^+^CD25^–^ effector T cells were sorted from 8- to 10-week-old female *Ptpn2^+/+^* CD45.2 SKG mice. For experiments evaluating the role of PTPN2 in cTregs (Figure 4, H–K; and Supplemental Figure 8), CD4^+^FoxP3^EGFP+^ were flow-sorted from the colon lamina propria of 8- to 10-week-old female CD45.2 FoxP3^EGFP^ *Ptpn2^+/+^* or *Ptpn2*^+/–^ SKG mice, and CD4^+^CD25^–^ effector T cells were sorted from 8- to 10-week-old female *Ptpn2^+/+^* CD45.1 SKG mice. For experiments evaluating the role of PTPN2 in GPR15^+^ and GPR15^–^ Tregs (Figure 5, E–H), GPR15^+^ or GPR15^–^ CD4^+^FoxP3^EGFP+^ SKG Tregs were sorted from pooled spleen and colonic lamina propria obtained from 8- to 10-week-old female CD45.2 FoxP3^EGFP^ *Ptpn2^+/+^* or *Ptpn2*^+/–^ SKG mice, and CD4^+^CD25^–^ effector T cells were sorted from 8- to 10-week-old female *Ptpn2^+/+^* CD45.1 SKG mice. After isolation, sorted Tregs (1 × 10^5^) were transferred in combination with CD4^+^CD25^–^ SKG effector T cells (4 × 10^5^) to 8-week-old female Rag2-KO mice. One week after transfer, mice were injected i.p. with 20 mg of mannan to boost induction of arthritis. Development of arthritis in littermate mice was evaluated in a blinded manner by clinical scoring and measurement of ankle thickness as described above. At the end of the experiment, flow cytometry was used for analysis of CD4^+^ T cells in lymph nodes, ankles, and colons of arthritic mice.

### Assessment of Treg stability in fate-mapping mice.

Briefly, single-cell suspensions were prepared from spleens, lymph nodes, and colonic lamina propria obtained from B6.SKG.H2^d/d^ FoxP3^YFP-Cre+/–^ tdTom^fl/+^ *Ptpn2*^+/+^ or *Ptpn2*^fl/+^ or B6.SKG.H2^d/d^ FoxP3^EGFP-ERT2-Cre+/+^ tdTomato^fl/fl^ *Ptpn2^+/+^* or *Ptpn2*^fl/+^ female mice as described above. Isolated cells were then stimulated with PMA, ionomycin, and brefeldin A for 5 hours as described above. After stimulation the presence of IL-17^+^ exTregs was analyzed by flow cytometry.

### Treg suppression assay in vitro.

GPR15^+^ or GPR15^–^ Tregs (CD4^+^FoxP3^EGFP+^) were flow-sorted from pooled spleens and colonic lamina propria isolated from either *Ptpn2*^+/+^ or *Ptpn2*^+/–^ 8-week-old BALB/c FoxP3^EGFP^ SKG mice. Naive CD4^+^ T cells (CD4^+^CD62L^+^CD44^lo^CD25^–^) were sorted from the spleens of BALB/c *Ptpn2*^+/+^ mice. Naive CD4^+^ T cells were stained with CellTrace Violet (C34557, Invitrogen, Thermo Fisher Scientific) to track proliferation. CellTrace-labeled naive CD4^+^ T cells (5 × 10^4^) were then cocultured with varying numbers of Tregs, in the presence of 5 μg/mL of soluble anti-CD3 (100302, BioLegend) and 1 × 10^5^ units of mytomycin C–treated (60 μg, M4287, MilliporeSigma) splenocytes from female BALB/c Rag2-KO mice as antigen-presenting cells. Cells were cultured for 3 days, after which proliferation of naive CD4^+^ T cells was evaluated by flow cytometry.

### Statistics.

Sample sizes were selected based on our experience with the abovementioned assays in order to achieve sufficient power to detect biologically relevant differences in the experiments being conducted. For statistical analysis, 2-tailed Mann-Whitney *U* test was performed on nonparametric data, and 2-tailed unpaired *t* test was performed on normally distributed data. For comparison of multiple parameters, 1-way or 2-way ANOVA was used. The statistical test used for each individual experiment is reported in the figure legend. All statistical calculations were performed using GraphPad Prism software. A comparison was considered significant if *P* was less than 0.05.

### Study approval.

The studies in animals were conducted in accordance with a protocol approved by the IACUC of the UCSD (protocol S16098).

## Author contributions

WCH, MNDS, SMS, and NB designed experiments. MLT and SS provided critical mouse models and reagents. WCH, MNDS, and MZ performed experiments. WCH, MNDS, SMS, MZ, and NB analyzed data. WCH, MNDS, and NB wrote the manuscript. All authors reviewed and approved the final version of the manuscript.

## Supplementary Material

supplemental data

## Version 1 10/15/2020

Electronic publication

## References

[1] Terao C, Raychaudhuri S, Gregersen PK (2016). Recent advances in defining the genetic basis of rheumatoid arthritis. Annu Rev Genomics Hum Genet.

[2] Viatte S, Plant D, Raychaudhuri S (2013). Genetics and epigenetics of rheumatoid arthritis. Nat Rev Rheumatol.

[3] Costantino F, Breban M, Garchon HJ (2018). Genetics and functional genomics of spondyloarthritis. Front Immunol.

[4] Westra HJ (2018). Fine-mapping and functional studies highlight potential causal variants for rheumatoid arthritis and type 1 diabetes. Nat Genet.

[5] Smolen JS (2018). Rheumatoid arthritis. Nat Rev Dis Primers.

[6] Gracey E (2020). Revisiting the gut-joint axis: links between gut inflammation and spondyloarthritis. Nat Rev Rheumatol.

[7] Scher JU, Abramson SB (2013). Periodontal disease, Porphyromonas gingivalis, and rheumatoid arthritis: what triggers autoimmunity and clinical disease?. Arthritis Res Ther.

[8] Horta-Baas G, Romero-Figueroa MDS, Montiel-Jarquín AJ, Pizano-Zárate ML, García-Mena J, Ramírez-Durán N (2017). Intestinal dysbiosis and rheumatoid arthritis: a link between gut microbiota and the pathogenesis of rheumatoid arthritis. J Immunol Res.

[9] Maeda Y, Takeda K (2019). Host-microbiota interactions in rheumatoid arthritis. Exp Mol Med.

[10] Scher JU (2013). Expansion of intestinal Prevotella copri correlates with enhanced susceptibility to arthritis. Elife.

[11] Pianta A (2017). Two rheumatoid arthritis-specific autoantigens correlate microbial immunity with autoimmune responses in joints. J Clin Invest.

[12] Zhang X (2015). The oral and gut microbiomes are perturbed in rheumatoid arthritis and partly normalized after treatment. Nat Med.

[13] Smith PM (2013). The microbial metabolites, short-chain fatty acids, regulate colonic Treg cell homeostasis. Science.

[14] Whibley N, Tucci A, Powrie F (2019). Regulatory T cell adaptation in the intestine and skin. Nat Immunol.

[15] Salmi M, Rajala P, Jalkanen S (1997). Homing of mucosal leukocytes to joints. Distinct endothelial ligands in synovium mediate leukocyte-subtype specific adhesion. J Clin Invest.

[16] Teng F (2016). Gut microbiota drive autoimmune arthritis by promoting differentiation and migration of Peyer’s patch T follicular helper cells. Immunity.

[17] Naskar D, Teng F, Felix KM, Bradley CP, Wu HJ (2017). Synthetic retinoid AM80 ameliorates lung and arthritic autoimmune responses by inhibiting T follicular helper and Th17 cell responses. J Immunol.

[18] Wu HJ (2010). Gut-residing segmented filamentous bacteria drive autoimmune arthritis via T helper 17 cells. Immunity.

[19] Asquith DL, Miller AM, McInnes IB, Liew FY (2009). Animal models of rheumatoid arthritis. Eur J Immunol.

[20] Jubair WK (2018). Modulation of Inflammatory Arthritis in Mice by Gut Microbiota Through Mucosal Inflammation and Autoantibody Generation. Arthritis Rheumatol.

[21] Rehaume LM (2014). ZAP-70 genotype disrupts the relationship between microbiota and host, leading to spondyloarthritis and ileitis in SKG mice. Arthritis Rheumatol.

[22] Maeda Y (2016). Dysbiosis contributes to arthritis development via activation of autoreactive T cells in the intestine. Arthritis Rheumatol.

[23] Sakaguchi N (2003). Altered thymic T-cell selection due to a mutation of the ZAP-70 gene causes autoimmune arthritis in mice. Nature.

[24] Benham H (2014). Interleukin-23 mediates the intestinal response to microbial beta-1,3-glucan and the development of spondyloarthritis pathology in SKG mice. Arthritis Rheumatol.

[25] Lees CW, Barrett JC, Parkes M, Satsangi J (2011). New IBD genetics: common pathways with other diseases. Gut.

[26] Thompson SD (2010). The susceptibility loci juvenile idiopathic arthritis shares with other autoimmune diseases extend to PTPN2, COG6, and ANGPT1. Arthritis Rheum.

[27] Richard-Miceli C, Criswell LA (2012). Emerging patterns of genetic overlap across autoimmune disorders. Genome Med.

[28] Okada Y (2012). Meta-analysis identifies nine new loci associated with rheumatoid arthritis in the Japanese population. Nat Genet.

[29] Freudenberg J (2011). Genome-wide association study of rheumatoid arthritis in Koreans: population-specific loci as well as overlap with European susceptibility loci. Arthritis Rheum.

[30] Cobb JE (2013). Identification of the tyrosine-protein phosphatase non-receptor type 2 as a rheumatoid arthritis susceptibility locus in europeans. PLoS One.

[31] Spalinger MR, McCole DF, Rogler G, Scharl M (2016). Protein tyrosine phosphatase non-receptor type 2 and inflammatory bowel disease. World J Gastroenterol.

[32] Long SA (2011). An autoimmune-associated variant in PTPN2 reveals an impairment of IL-2R signaling in CD4(+) T cells. Genes Immun.

[33] Scharl M (2012). Crohn’s disease-associated polymorphism within the PTPN2 gene affects muramyl-dipeptide-induced cytokine secretion and autophagy. Inflamm Bowel Dis.

[34] Scharl M (2012). Protein tyrosine phosphatase nonreceptor type 2 regulates autophagosome formation in human intestinal cells. Inflamm Bowel Dis.

[35] Heinonen KM, Bourdeau A, Doody KM, Tremblay ML (2009). Protein tyrosine phosphatases PTP-1B and TC-PTP play nonredundant roles in macrophage development and IFN-gamma signaling. Proc Natl Acad Sci U S A.

[36] Doody KM, Bourdeau A, Tremblay ML (2009). T-cell protein tyrosine phosphatase is a key regulator in immune cell signaling: lessons from the knockout mouse model and implications in human disease. Immunol Rev.

[37] You-Ten KE (1997). Impaired bone marrow microenvironment and immune function in T cell protein tyrosine phosphatase-deficient mice. J Exp Med.

[38] Wiede F (2011). T cell protein tyrosine phosphatase attenuates T cell signaling to maintain tolerance in mice. J Clin Invest.

[39] Wiede F, La Gruta NL, Tiganis T (2014). PTPN2 attenuates T-cell lymphopenia-induced proliferation. Nat Commun.

[40] Simoncic PD, Lee-Loy A, Barber DL, Tremblay ML, McGlade CJ (2002). The T cell protein tyrosine phosphatase is a negative regulator of janus family kinases 1 and 3. Curr Biol.

[41] Pike KA, Tremblay ML (2016). TC-PTP and PTP1B: Regulating JAK-STAT signaling, controlling lymphoid malignancies. Cytokine.

[42] LaFleur MW (2019). PTPN2 regulates the generation of exhausted CD8^+^ T cell subpopulations and restrains tumor immunity. Nat Immunol.

[43] Wiede F (2020). PTPN2 phosphatase deletion in T cells promotes anti-tumour immunity and CAR T-cell efficacy in solid tumours. EMBO J.

[44] Svensson MN (2019). Reduced expression of phosphatase PTPN2 promotes pathogenic conversion of Tregs in autoimmunity. J Clin Invest.

[45] Hassan SW, Doody KM, Hardy S, Uetani N, Cournoyer D, Tremblay ML (2010). Increased susceptibility to dextran sulfate sodium induced colitis in the T cell protein tyrosine phosphatase heterozygous mouse. PLoS One.

[46] Spalinger MR (2015). PTPN2 controls differentiation of CD4+T cells and limits intestinal inflammation and intestinal dysbiosis. Mucosal Immunol.

[47] Komatsu N, Takayanagi H (2015). Arthritogenic T cells in autoimmune arthritis. Int J Biochem Cell Biol.

[48] Firestein GS, McInnes IB (2017). Immunopathogenesis of rheumatoid arthritis. Immunity.

[49] van Hamburg JP, Tas SW (2018). Molecular mechanisms underpinning T helper 17 cell heterogeneity and functions in rheumatoid arthritis. J Autoimmun.

[50] Dominguez-Villar M, Raddassi K, Danielsen AC, Guarnaccia J, Hafler DA (2019). Fingolimod modulates T cell phenotype and regulatory T cell plasticity in vivo. J Autoimmun.

[51] Komatsu N (2014). Pathogenic conversion of Foxp3+ T cells into TH17 cells in autoimmune arthritis. Nat Med.

[52] Ruutu M (2012). β-glucan triggers spondylarthritis and Crohn’s disease-like ileitis in SKG mice. Arthritis Rheum.

[53] Omenetti S, Pizarro TT (2015). The Treg/Th17 axis: a dynamic balance regulated by the gut microbiome. Front Immunol.

[54] Sefik E (2015). Mucosal immunology. Individual intestinal symbionts induce a distinct population of RORγ+ regulatory T cells. Science.

[55] Ohnmacht C (2015). Mucosal immunology. The microbiota regulates type 2 immunity through RORγt+ T cells. Science.

[56] Ivanov (2006). The orphan nuclear receptor RORgammat directs the differentiation program of proinflammatory IL-17+ T helper cells. Cell.

[57] Dienz O, Rincon M (2009). The effects of IL-6 on CD4 T cell responses. Clin Immunol.

[58] Chassaing B, Aitken JD, Malleshappa M, Vijay-Kumar M (2014). Dextran sulfate sodium (DSS)-induced colitis in mice. Curr Protoc Immunol.

[59] Kim SV (2013). GPR15-mediated homing controls immune homeostasis in the large intestine mucosa. Science.

[60] Tanoue T, Atarashi K, Honda K (2016). Development and maintenance of intestinal regulatory T cells. Nat Rev Immunol.

[61] Nguyen LP (2015). Role and species-specific expression of colon T cell homing receptor GPR15 in colitis. Nat Immunol.

[62] Kim SV (2013). GPR15-mediated homing controls immune homeostasis in the large intestine mucosa. Science.

[63] Kim BS (2017). Generation of RORγt^+^ antigen-specific T regulatory 17 cells from Foxp3^+^ precursors in autoimmunity. Cell Rep.

[64] Firestein GS (2009). Rheumatoid arthritis in a mouse?. Nat Clin Pract Rheumatol.

[65] Bilsborough J, Viney JL (2015). GPR15: a tale of two species. Nat Immunol.

[66] McCole DF (2012). Regulation of epithelial barrier function by the inflammatory bowel disease candidate gene, PTPN2. Ann N Y Acad Sci.

[67] Stuart PE (2015). Genome-wide association analysis of psoriatic arthritis and cutaneous psoriasis reveals differences in their genetic architecture. Am J Hum Genet.

[68] McGonagle DG, McInnes IB, Kirkham BW, Sherlock J, Moots R (2019). The role of IL-17A in axial spondyloarthritis and psoriatic arthritis: recent advances and controversies. Ann Rheum Dis.

[69] Hashimoto M (2010). Complement drives Th17 cell differentiation and triggers autoimmune arthritis. J Exp Med.

[70] Bussières-Marmen S, Vinette V, Gungabeesoon J, Aubry I, Pérez-Quintero LA, Tremblay ML (2018). Loss of T-cell protein tyrosine phosphatase in the intestinal epithelium promotes local inflammation by increasing colonic stem cell proliferation. Cell Mol Immunol.

[71] Rubtsov YP (2008). Regulatory T cell-derived interleukin-10 limits inflammation at environmental interfaces. Immunity.

[72] Rubtsov YP (2010). Stability of the regulatory T cell lineage in vivo. Science.

[73] Madisen L (2010). A robust and high-throughput Cre reporting and characterization system for the whole mouse brain. Nat Neurosci.

[74] Lin W (2007). Regulatory T cell development in the absence of functional Foxp3. Nat Immunol.

[75] van der Veeken J (2016). Memory of inflammation in regulatory T cells. Cell.

[76] Guma M, Ronacher L, Liu-Bryan R, Takai S, Karin M, Corr M (2009). Caspase 1-independent activation of interleukin-1beta in neutrophil-predominant inflammation. Arthritis Rheum.

[77] Obermeier F, Kojouharoff G, Hans W, Schölmerich J, Gross V, Falk W (1999). Interferon-gamma (IFN-gamma)- and tumour necrosis factor (TNF)-induced nitric oxide as toxic effector molecule in chronic dextran sulphate sodium (DSS)-induced colitis in mice. Clin Exp Immunol.

[78] Seo GY (2018). LIGHT-HVEM signaling in innate lymphoid cell subsets protects against enteric bacterial infection. Cell Host Microbe.

