# Supplementary material for: PTPN2 links colonic and joint inflammation in experimental autoimmune arthritis
Source: JCI Insight. 2020 Oct 15;5(20):e141868. doi: 10.1172/jci.insight.141868 (PMC7605542; doi:10.1172/jci.insight.141868)
Supplement: supplemental data [file jciinsight-5-141868-s154.pdf]

# Supplemental Material

# PTPN2 links colonic and joint inflammation in experimental autoimmune arthritis

Wan-Chen Hsieh[1], Mattias N.D. Svensson[1, 2], Martina Zoccheddu[1], Michael L. Tremblay[3,4,5], Shimon Sakaguchi[6,7], Stephanie M. Stanford[1], and Nunzio Bottini[1]

[1]Department of Medicine, UCSD School of Medicine, La Jolla, California, USA. [2]Department of Rheumatology and Inflammation Research, Sahlgrenska Academy, University of Gothenburg, Gothenburg, Sweden.
[3]Rosalind and Morris Goodman Cancer Research Centre, [4]Department of Biochemistry, and [5]Division of Experimental Medicine, Department of Medicine, Faculty of Medicine and Health Sciences, McGill University, Montréal, Québec, Canada.
[6]Laboratory of Experimental Immunology, Immunology Frontier Research Center, Osaka University, Suita, Japan.
[7]Department of Experimental Pathology, Institute for Frontier Medical Sciences, Kyoto University, Kyoto, Japan.

Address correspondence to: Nunzio Bottini, University of California, San Diego, Department of Medicine, 9500 Gilman Drive, MC0656, La Jolla, California, USA.
Phone: 858.246.2398; Email: nbottini@ health.ucsd.edu.

## Supplemental Figure 1

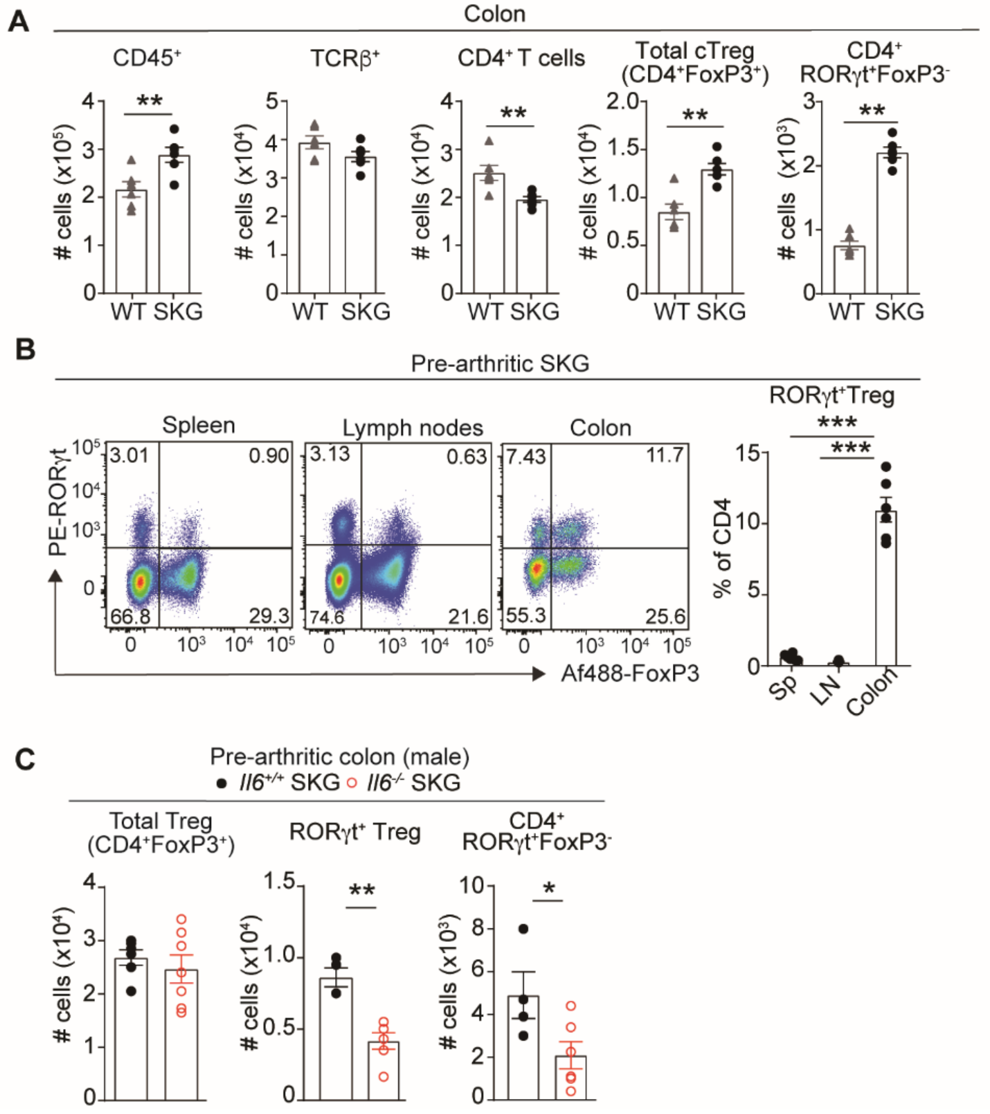


**Supplemental Figure 1. Additional T cell counts in mice assessed in Figure 1.** (**A**) Number of leukocytes (CD45$^+$), total T cells (TCRβ$^+$), CD4$^+$ T cells (TCRβ$^+$CD4$^+$), total Tregs (TCRβ$^+$CD4$^+$FoxP3$^+$) and RORγt$^+$FoxP3$^-$CD4$^+$ T cells (CD45$^+$TCRβ$^+$CD4$^+$RORγt$^+$ FoxP3$^-$) in colon isolated from mice in **Figure 1A**. (**B**) Representative flow cytometric staining for FoxP3- and RORγt-expressing CD4$^+$ T cells in spleen, lymph nodes and colons of pre-arthritic SKG mice (n=7). (**C**) Number of total Tregs (CD4$^+$FoxP3$^+$), RORγt$^+$ Tregs (CD4$^+$FoxP3$^+$RORγt$^+$) and RORγt$^+$FoxP3$^-$ CD4$^+$ T cells (CD4$^+$FoxP3$^-$RORγt$^+$) in colons of pre-arthritic male *Il6*$^{+/+}$ (n=6) and *Il6*$^{-/-}$ (n=7) SKG mice. Each symbol in **A-C** represents an individual mouse. Graphs show mean ± SEM. $^{**}P < 0.01$, $^{***}P < 0.001$ by unpaired t-test (**A** and **C**) or 1-way ANOVA (**B**).

## Supplementary figure 2

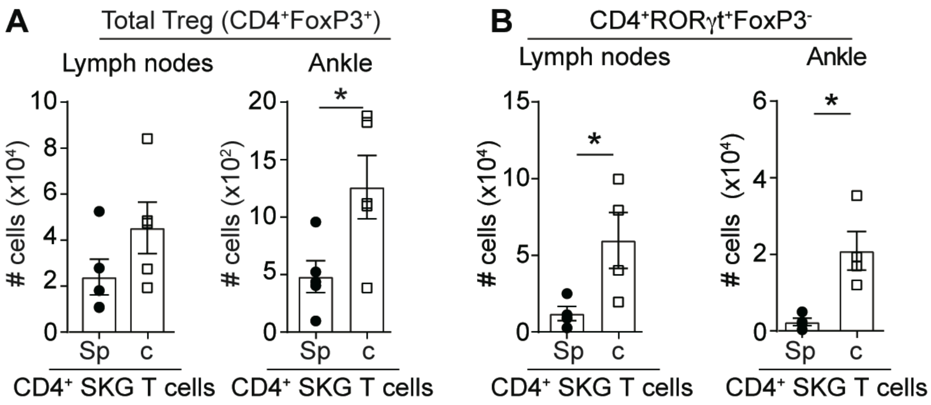


**Supplemental Figure 2. Total Tregs and ROR$\gamma$t$^{+}$FoxP3$^{-}$CD4$^{+}$ T cell counts in lymph nodes and ankles of arthritic Rag2-KO mice**. (**A-B**) Flow cytometric analysis of **Figure 1D-F**. (**A**) Total Tregs (TCR$\beta^{+}$CD4$^{+}$FoxP3$^{+}$) and (**B**) ROR$\gamma$t$^{+}$FoxP3$^{-}$ CD4$^{+}$ T cells (CD45$^{+}$TCR$\beta^{+}$CD4$^{+}$ROR$\gamma$t$^{+}$FoxP3$^{-}$) cells in lymph nodes and ankles of arthritic Rag2-KO mice. Compiled data from 2 independent experiments are shown. Each symbol represents an individual mouse. Graphs show mean ± SEM. *$P < 0.05$ by unpaired t-test.

## Supplemental Figure 3

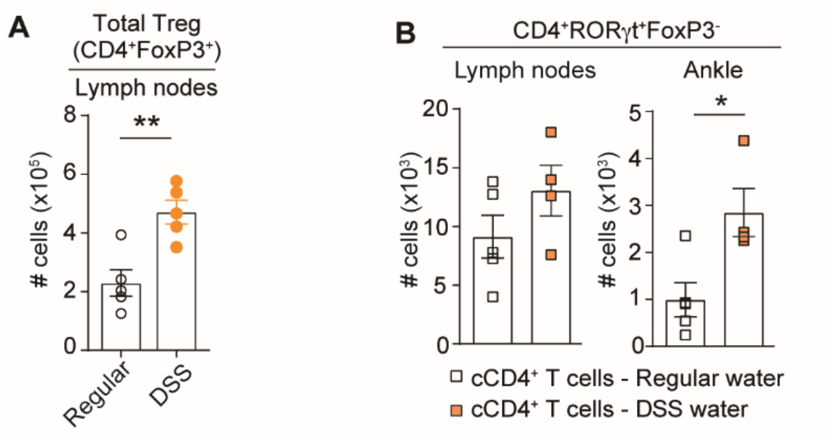


**Supplemental Figure 3. Total Tregs and RORγt$^+$FoxP3$^-$CD4$^+$ T cell counts in lymph nodes and ankles of DSS-induced arthritic mice**. (**A**) Number of total Tregs (CD45.1$^+$CD4$^+$FoxP3$^+$) in lymph nodes isolated from mice in **Figure 2A-E**. (**B**) Flow cytometric analysis of RORγt$^+$FoxP3$^-$CD4$^+$ T cells (CD4$^+$RORγt$^+$FoxP3$^-$) in lymph nodes and ankles of arthritic Rag2-KO mice in **Figure 2F-H**. Compiled data from 2 independent experiments are shown in **A**. Each symbol in **A** and **B** represents an individual mouse. Graphs show mean ± SEM. *$P$ < 0.05, **$P$ < 0.01 by unpaired t-test (**A** and **B**).

**Supplemental Figure 4**

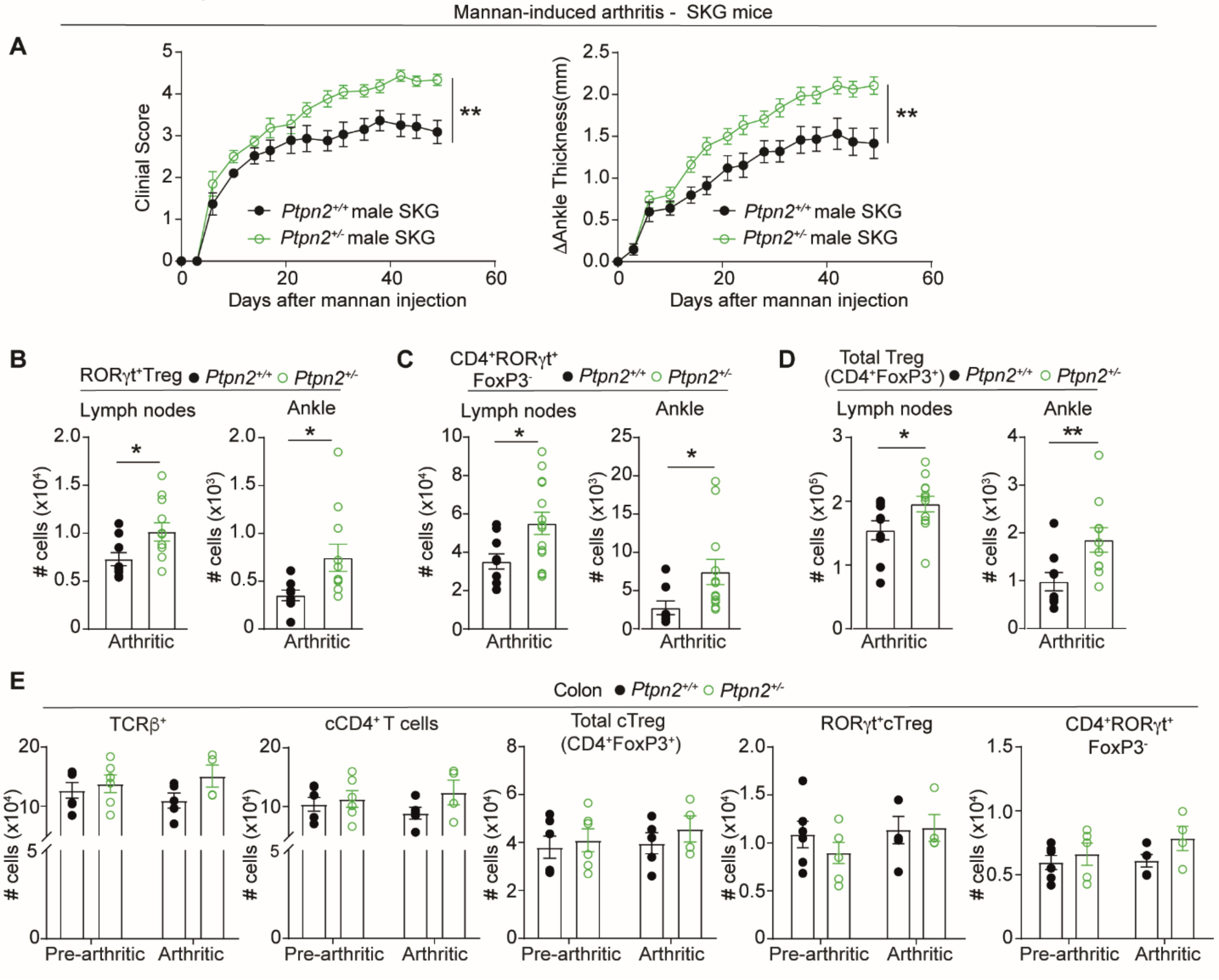


**Supplemental Figure 4. *Ptpn2* haploinsufficiency promotes mannan-induced arthritis in male SKG mice**. (**A-D**) Male *Ptpn2*$^{+/+}$ (n=9) and *Ptpn2*$^{+/-}$ (n=15) SKG mice were injected intraperitoneally with mannan and arthritis development was monitored by clinical scoring and measurement of ankle swelling. Mice were analyzed at day 49. (**A**) Clinical score and change in ankle thickness. (**B**) Number of RORγt$^{+}$ Tregs (CD4$^{+}$ FoxP3$^{+}$RORγt$^{+}$) in lymph nodes and ankles. (**C**) Number of RORγt$^{+}$FoxP3$^{-}$ CD4$^{+}$ T cells in lymph nodes and ankles. (**D**) Number of total Tregs (CD4$^{+}$FoxP3$^{+}$) in lymph nodes and ankles. (**E**) Number of T cells (TCRβ$^{+}$), CD4$^{+}$ T cells (TCRβ$^{+}$CD4$^{+}$), total Tregs (CD4$^{+}$FoxP3$^{+}$), RORγt$^{+}$ Tregs (CD4$^{+}$FoxP3$^{+}$RORγt$^{+}$) and RORγt$^{+}$FoxP3$^{-}$CD4$^{+}$ T cells in the colon of pre-arthritic or 10-days post mannan SKG mice (*Ptpn2*$^{+/+}$ (n=6) and *Ptpn2*$^{+/-}$ (n=6) pre-arthritic SKG mice; *Ptpn2*$^{+/+}$ (n=5) and *Ptpn2*$^{+/-}$ (n=4) SKG mice 10-days post mannan mice). Compiled data from 4 independent experiments are shown in **A-E**. Each symbol in **B-E** represents an individual mouse. Clinical score and swelling ankles were quantified using the area under the curve. Graphs show mean ± SEM. *$P$ < 0.05, **$P$ < 0.01 by Mann-Whitney $U$ test (**A**), unpaired t-test (**B-D**) or 2-way ANOVA (**E**).

## Supplemental Figure 5

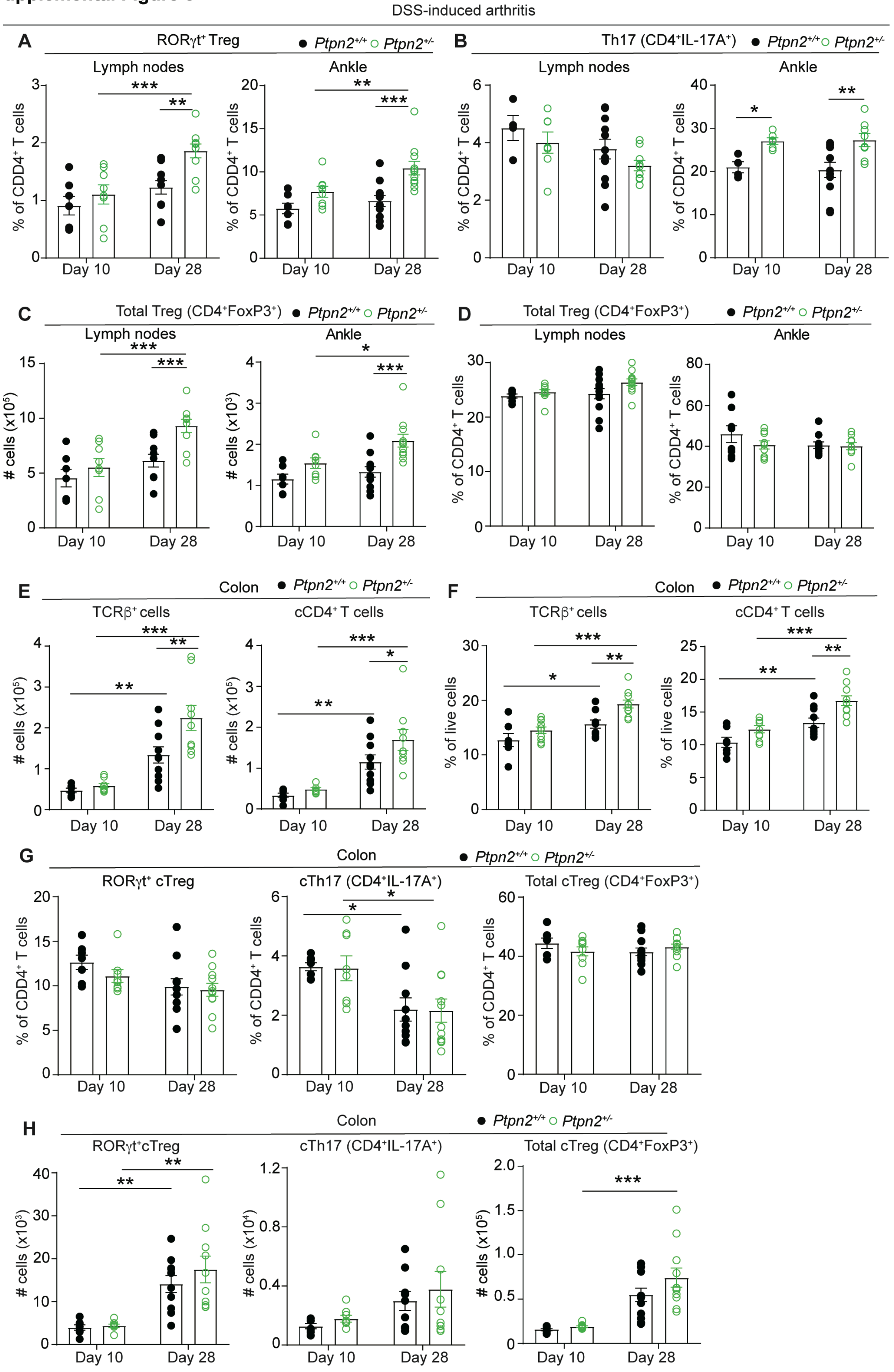

**Supplemental Figure 5. Frequency and number of total Tregs, RORγt-expressing Tregs, Th17, T cells and $CD4^+$ T cells in SKG mice with DSS-induced arthritis.**

(**A-H**) Cells isolated from each tissue 10 days or 28 days after start of 0.5% DSS water treatment of mice shown in **Figure 3**. (**A**) Frequency of $ROR\gamma t^+$ Tregs ($CD4^+FoxP3^+$ $ROR\gamma t^+$) in lymph nodes and ankles. (**B**) Frequency of Th17 ($CD4^+IL\text{-}17A^+FoxP3^-$) cells in lymph nodes and ankles. (**C-D**) Number (**C**) and frequency (**D**) of total Tregs ($CD4^+$ $FoxP3^+$) in lymph nodes and ankles. (**E-F**) Number (**E**) and frequency (**F**) of T cells ($TCR\beta^+$) and $CD4^+$ T cells ($TCR\beta^+CD4^+$) in colon. (**G-H**) Frequency (**G**) and number (**H**) of $ROR\gamma t^+$ cTregs ($CD4^+ROR\gamma t^+FoxP3^+$), cTh17 ($CD4^+IL\text{-}17A^+FoxP3^-$) and total cTregs ($CD4^+$ $FoxP3^+$) in colon. Compiled data from 6 independent experiments are shown in **A-H**.Each symbol represents an individual mouse. Graphs show mean ± SEM. $^*P < 0.05$, $^{**}P < 0.01$, $^{***}P < 0.005$ by 2-way ANOVA (**A-H**).

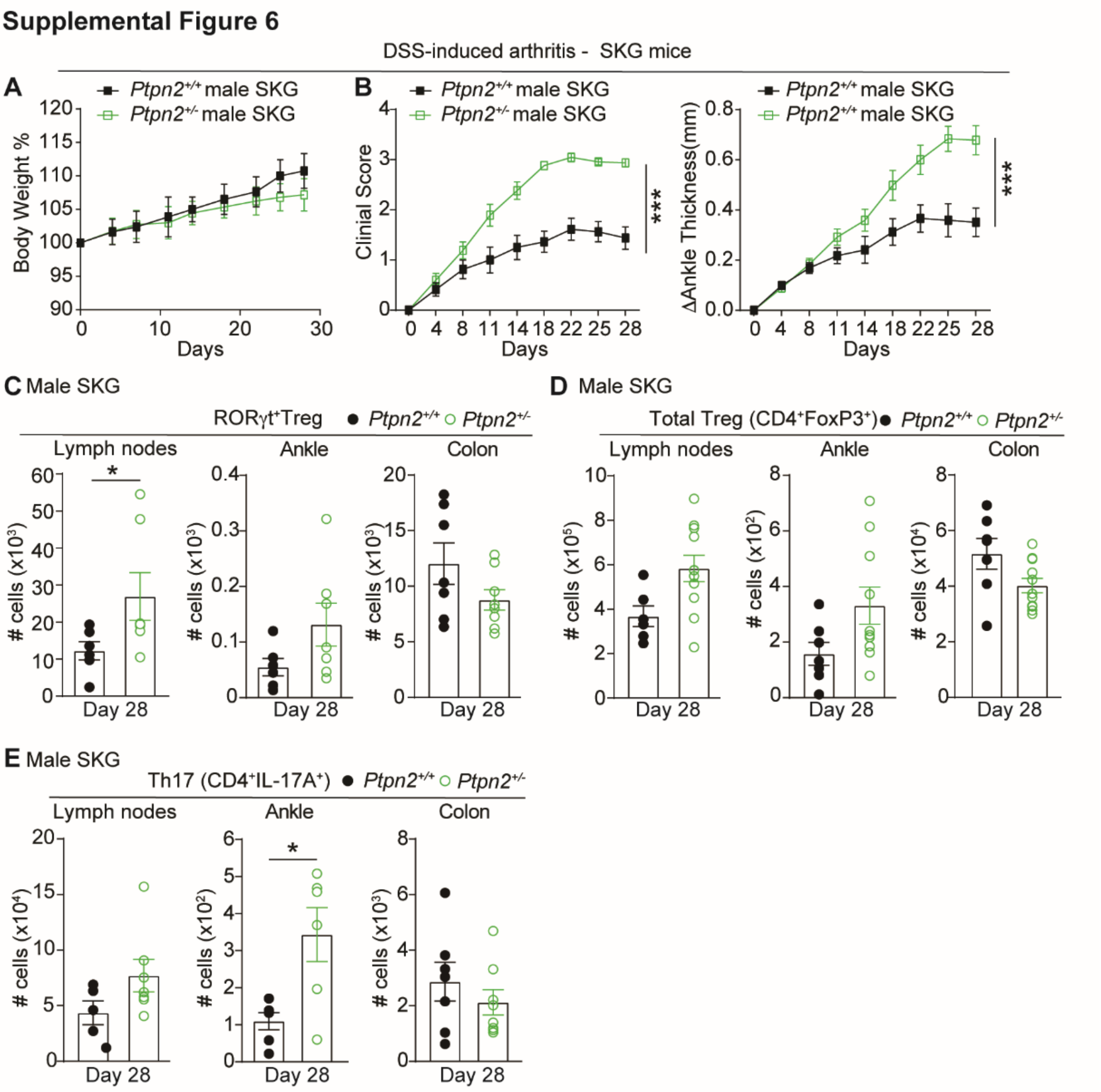


**Supplemental Figure 6. *Ptpn2* haploinsufficiency enhances susceptibility to DSS-induced arthritis in male SKG mice.** (**A-E**) Male *Ptpn2*$^{+/+}$ (n=8) and *Ptpn2*$^{+/-}$ (n=11) SKG mice received 0.5% DSS in their drinking water as in **Figure 3A**. (**A**) Change in body weight. (**B**) Clinical score and change in ankle thickness. (**C-E**) Number of RORγt$^{+}$ Tregs (**C**; CD4$^{+}$FoxP3$^{+}$RORγt$^{+}$), total Tregs (**D**; CD4$^{+}$FoxP3$^{+}$) and Th17 (**E**; CD4$^{+}$IL-17A$^{+}$FoxP3$^{-}$) in lymph nodes, ankles, and colon. Compiled data from 7 independent experiments are shown in **A-E**. Each symbol in **C-E** represents an individual mouse. Arthritis severity was quantified using the area under the curve. Graphs show mean ± SEM. **P* < 0.05, ****P* < 0.001 by Mann-Whitney *U* test (**B**), or unpaired t-test (**C-E**).

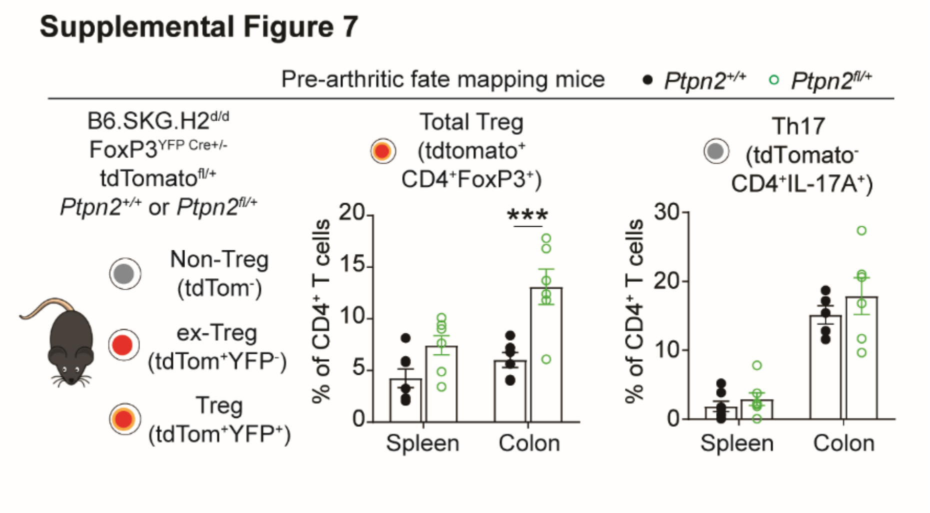


**Supplemental Figure 7. Frequency of total Tregs and Th17 in pre-arthritic fate-mapping SKG mice**. Frequency of total Tregs ($CD4^{+}tdTomato^{+}FoxP3^{YFP+}$) and tdTomato- Th17 ($CD4^{+}tdTomato^{-}IL$-$17A^{+}FoxP3^{YFP-}$) in spleen and colon isolated from mice shown in **Figure 4A**. Compiled data from 2 independent experiments are shown. Each symbol represents an individual mouse. Graphs show mean ± SEM**.** ***$P < 0.001$ by unpaired t-test.

**Supplemental Figure 8**

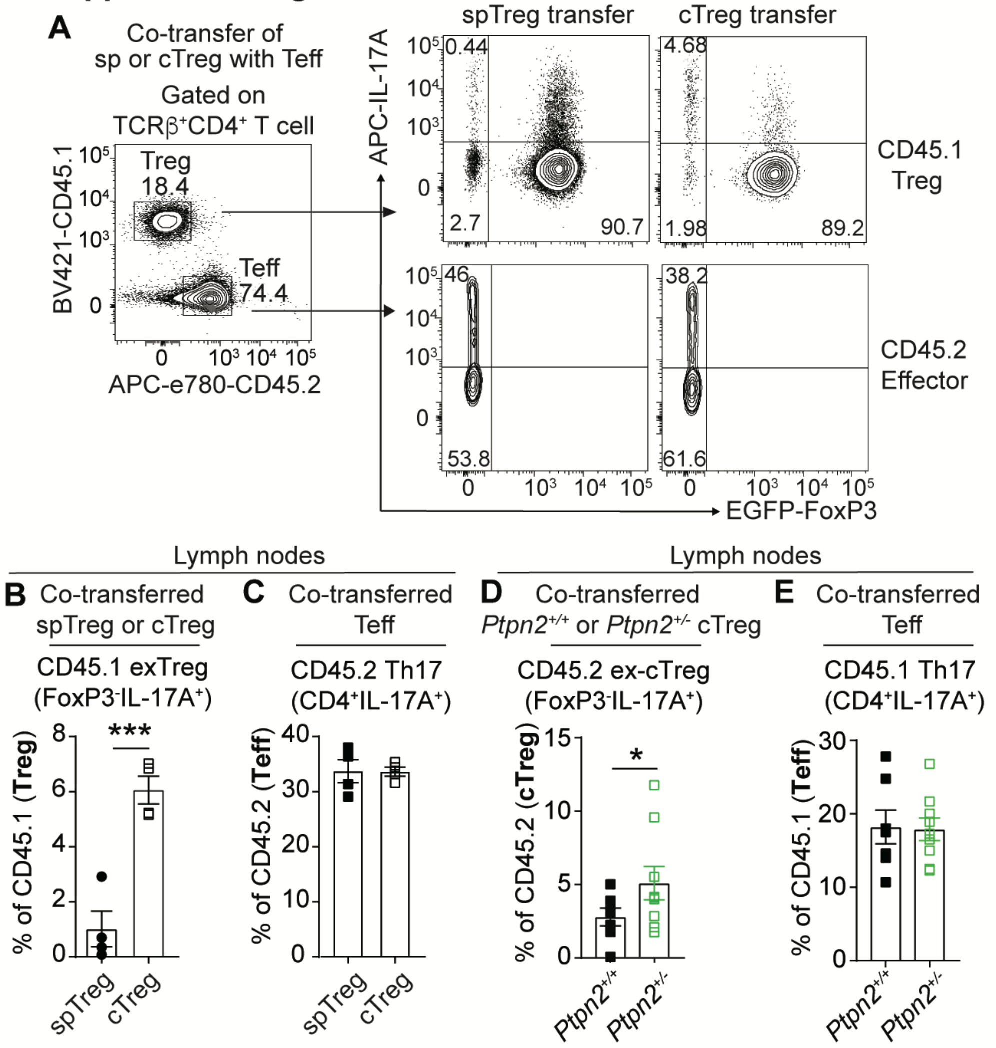


**Supplemental Figure 8. SKG cTregs are more arthritogenic than SKG spTregs after transfer into Rag2-KO mice**. (**A-C**) Analysis of transferred Tregs and Teff by flow cytometry from recipient Rag2-KO mice in **Figure 4D-E** receiving either SKG splenic (sp) or colonic (c) Tregs. (**A**) Representative gating strategy. (**B**) Frequency of exTregs (CD45.1$^+$CD4$^+$IL-17$^+$FoxP3$^-$) in lymph nodes of recipient mice. (**C**) Frequency of Th17 (CD4$^+$IL17A$^+$FoxP3$^-$) among transferred CD45.2 SKG CD4$^+$CD25$^-$ Teffs in lymph nodes of recipient mice. (**D-E**) Analysis of transferred Tregs and Teff by flow cytometry from recipient Rag2-KO mice in **Figure 4H-I** receiving cTregs from *Ptpn2*$^{+/+}$ or *Ptpn2*$^{+/-}$ SKG mice. (**D**) Frequency of *Ptpn2*$^{+/+}$ and *Ptpn2*$^{+/-}$ ex-cTreg (CD45.2$^+$CD4$^+$IL-17$^+$FoxP3$^-$) cells in lymph nodes of recipient mice. (**E**) Frequency of Th17 cells (CD4$^+$IL17A$^+$FoxP3$^-$) among transferred CD45.1 SKG CD4$^+$CD25$^-$ *Ptpn2*$^{+/+}$ (WT) Teffs in lymph nodes of recipient mice. Compiled data from 2 independent experiments are shown in **B-E**. Each symbol in **B-E** represents an individual mouse. Graphs show mean ± SEM. **P* < 0.05, ****P* < 0.001 by unpaired t-test.

**Supplemental Figure 9**

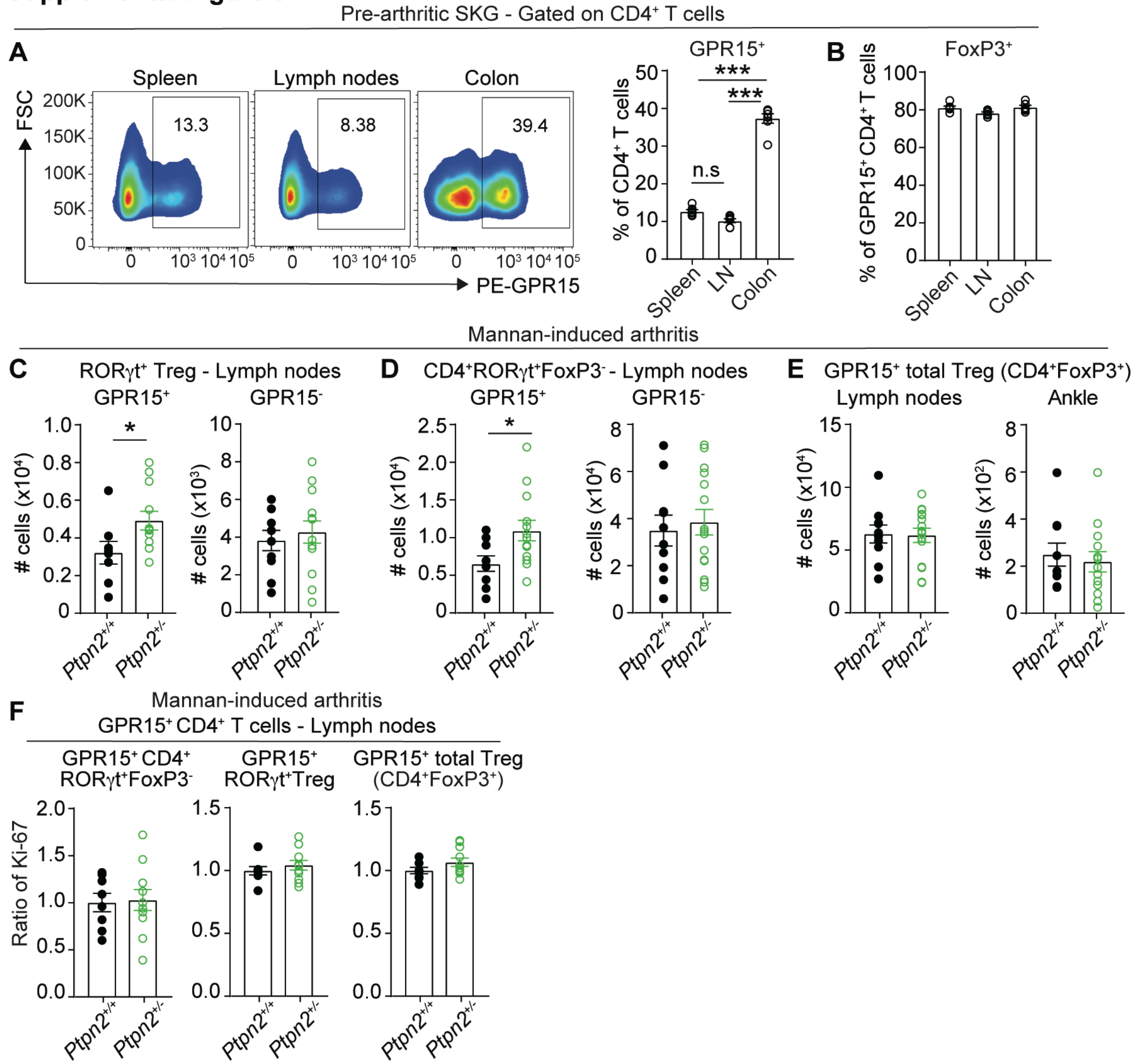


**Supplemental Figure 9. Enrichment of GPR15⁺ CD4⁺ T cells correlates with enhanced arthritis in *Ptpn2* haploinsufficient SKG mice.** (**A**) Representative gating (*left*) and frequency (*right*) of GPR15⁺ expressing cells among CD4⁺ T cells in spleen (Sp), lymph nodes (LN) and colon of pre-arthritic SKG mice (n=7). (**B**) Frequency of FoxP3⁺ cells among GPR15⁺ CD4⁺ T cells in 9 week-old pre-arthritic SKG mice (n=7). (**C-F**) Cells isolated from *Ptpn2*$^{+/+}$ (n=9) and *Ptpn2*$^{+/-}$ (n=13) SKG mice with mannan-induced arthritis from **Supplemental Figure 4**. Mice were analyzed at day 49 after mannan injection. (**C**) Number of GPR15⁺ and GPR15⁻ RORγt⁺ Tregs (CD4⁺FoxP3⁺RORγt⁺) in lymph nodes. (**D**) Number of GPR15⁺ and GPR15⁻ RORγt⁺FoxP3⁻CD4 T cells (CD4⁺FoxP3⁻RORγt⁺ GPR15⁺ or GPR15⁻) in lymph nodes. (**E**) Number of GPR15⁺ total Tregs (CD4⁺FoxP3⁺GPR15⁺) in lymph nodes and ankles. (**F**) Ki-67 MFI ratio in GPR15-expressing RORγt⁺FoxP3⁻CD4⁺ T cells (CD4⁺FoxP3⁻RORγt⁺GPR15⁺), RORγt⁺ Tregs (CD4⁺RORγt⁺FoxP3⁺GPR15⁺) and total Tregs (CD4⁺FoxP3⁺GPR15⁺) in lymph nodes. Compiled data from at least 2 independent experiments are shown. Each symbol in **A-F** shows individual mice. Graphs show mean ± SEM. **P* < 0.05, ****P* < 0.005 by 1-way ANOVA (**A**) or unpaired t-test (**C-D**).

**Supplemental Figure 10**

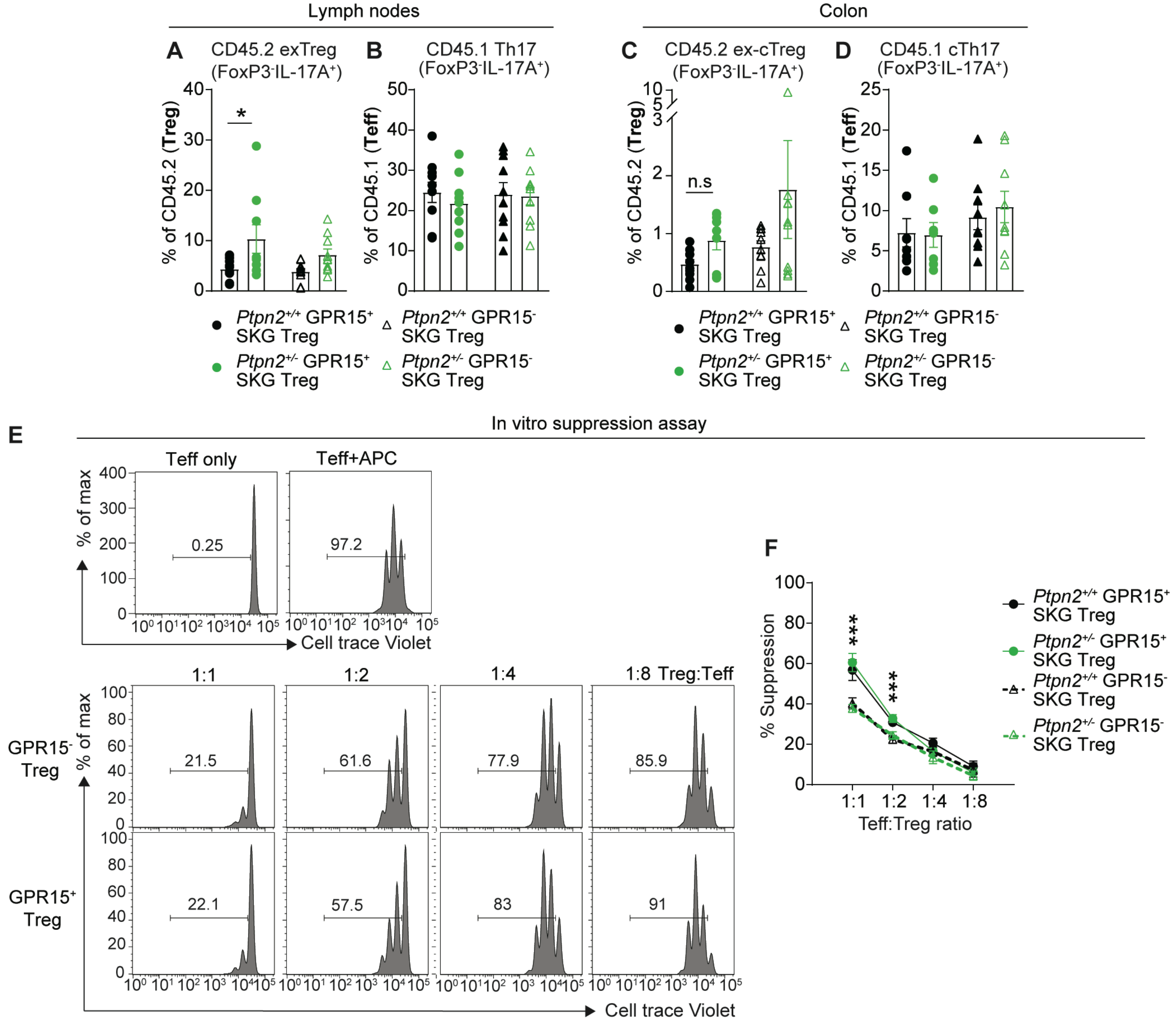


**Supplemental Figure 10. *Ptpn2* haploinsufficiency causes increased conversion of GRP15$^+$ SKG Tregs to IL-17$^+$ exTregs during arthritis.** (**A-D**) Frequency of IL-17 producing exTregs (CD45.2$^+$CD4$^+$IL-17A$^+$FoxP3$^-$) and Th17 (CD45.1$^+$CD4$^+$IL-17A$^+$FoxP3$^-$) in lymph nodes (**A-B**) and colon (**C-D**) of Rag2-KO recipient mice shown in **Figure 5E-H**. (**E-F**) Percent suppression of naïve CD4$^+$ T cell proliferation in vitro by GPR15$^+$ and GPR15$^-$ Tregs isolated from either *Ptpn2*$^{+/+}$ (n=3) or *Ptpn2*$^{+/-}$ (n=3) FoxP3$^{EGFP}$ SKG mice. (**E**) Representative gating strategy for analysis of in vitro suppression assay by flow cytometry in (**F**). Compiled data from 3 independent experiments are shown. Each symbol in **A-D** represents an individual mouse. Graphs show mean ± SEM. *$P < 0.05$, ***$P < 0.001$ by 2-way ANOVA (**A**) unpaired t-test (**F**). Non-significant (n.s).

**Supplemental Figure 11**

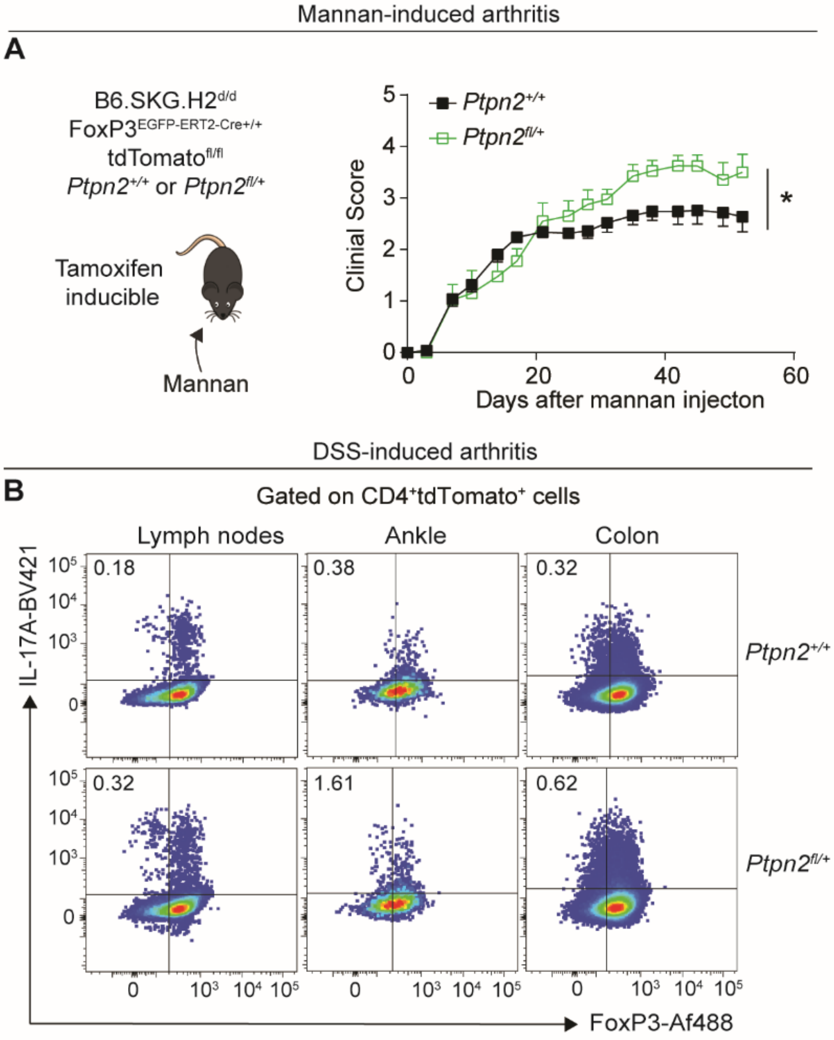


**Supplemental Figure 11. Inducible Treg-specific *Ptpn2* haploinsufficiency aggravates severity of DSS-induced arthritis.** (**A**) Clinical score of female B6.SKG.H2$^{d/d}$ FoxP3$^{EGFP\text{-}ERT2\text{-}Cre+/+}$ tdTomato$^{fl/fl}$ *Ptpn2*$^{+/+}$ (n=5) or *Ptpn2*$^{fl/+}$ (n=4) inducible Treg fate-mapping mice after intraperitoneal injection of mannan. Treg fate-mapping mice were administered 100 µL tamoxifen (20 mg/ml) via i.p. injection for 5 consecutive days to induce Cre expression. After the last treatment with tamoxifen, mice were rested for 2 weeks before induction of arthritis by mannan injection. (**B**) Representative flow cytometric gating of IL-17 producing cells among tdTomato$^{+}$ CD4$^{+}$ T cells isolated from B6.SKG.H2$^{d/d}$ FoxP3$^{EGFP\text{-}ERT2\text{-}Cre+/+}$ tdTomato$^{fl/fl}$ *Ptpn2*$^{+/+}$ or *Ptpn2*$^{fl/+}$ inducible Treg fate-mapping mice shown in **Figure 6**. Clinical score was quantified using the area under the curve. Graphs shows mean ± SEM. *$P < 0.05$ by Mann-Whitney $U$ test (**A**).
